# An Erg11 lanosterol 14-α-demethylase-Arv1 complex is required for *Candida albicans* virulence

**DOI:** 10.1371/journal.pone.0235746

**Published:** 2020-07-17

**Authors:** Michelle L. Villasmil, Antonio Daniel Barbosa, Jessie Lee Cunningham, Symeon Siniossoglou, Joseph T. Nickels

**Affiliations:** 1 Institute of Metabolic Disorders, Genesis Biotechnology Group, Hamilton, New Jersey, United States of America; 2 Cambridge Institute for Medical Research, University of Cambridge, Cambridge, United Kingdom; 3 Rutgers Center for Lipid Research, New Jersey Institute for Food, Nutrition, and Health, Rutgers University, New Brunswick, New Jersey, United States of America; Tulane University Health Sciences Center, UNITED STATES

## Abstract

Azole resistant fungal infections remain a health problem for the immune compromised. Current therapies are limited due to rises in new resistance mechanisms. Therefore, it is important to identify new drug targets for drug discovery and novel therapeutics. Arv1 (***a****re1 are2* **r**equired for **v**iability **1**) function is highly conserved between multiple pathogenic fungal species. *Candida albicans* (*C*. *albicans*) cells lacking CaArv1 are azole hypersusceptible and lack virulence. *Saccharomyces cerevisiae* (*S*. *cerevisiae*) *Scarv1* cells are also azole hypersusceptible, a phenotype reversed by expression of CaArv1, indicating conservation in the molecular mechanism for azole susceptibility. To define the relationship between Arv1 function and azole susceptibility, we undertook a structure/function analysis of ScArv1. We identified several conserved amino acids within the Sc**A**rv1 **h**omology **d**omain (ScAhd) required for maintaining normal azole susceptibility. Erg11 lanosterol 14-α-demethylase is the rate-limiting enzyme in sterol biosynthesis and is the direct target of azole antifungals, so we used our ScArv1 mutants in order to explore the relationship between ScArv1 and ScErg11. Specific ScArv1 mutants ectopically expressed from a low copy plasmid were unable to restore normal azole susceptibility to *Scarv1* cells and had reduced Erg11 protein levels. Erg11 protein stability depended on its ability to form a heterodimeric complex with Arv1. Complex formation was required for maintaining normal azole susceptibility. *Scarv1* cells expressing orthologous CaArv1 mutants also had reduced CaErg11 levels, were unable to form a CaArv1-CaErg11 complex, and were azole hypersusceptible. *Scarv1* cells expressing CaArv1 mutants unable to interact with CaErg11 could not sustain proper levels of the azole resistant CaErg11^Y132F F145L^ protein. *Caarv1/Caarv1* cells expressing CaArv1 mutants unable to interact with CaErg11 were found to lack virulence using a disseminated candidiasis mouse model. Expressing CaErg11^Y132F F145L^ did not reverse the lack of virulence. We hypothesize that the role of Arv1 in Erg11-dependent azole resistance is to stabilize Erg11 protein level. Arv1 inhibition may represent an avenue for treating azole resistance.

## Introduction

Azole antifungal drug resistance continues to be a major morbidity factor for the immune compromised [1]. In addition to *C*. *albicans* infections, an increasing number of azole resistant non-*albicans Candida spp*. are being identified in the clinic [1]. These include *Candida glabrata*, *Candida parapsilosis*, *Candida krusei*, and *Candida tropicalis* [2]. While the number of fungal infections attributed to *C*. *albicans* has plateaued, infections caused by these other *Candida spp*. continue to rise. It has been estimated that ~30–60% of azole resistant fungal infections are caused by non-*albicans Candida spp*. [3, 4], underscoring the need to identify alternative ways to treat azole resistant fungi.

The yeast ergosterol biosynthetic pathway is highly conserved, composed of almost 30 genes, and is housed within the ER [5]. The elegant work of the Bard laboratory strongly suggests that a large ER-localized ergosome complex exists, which harbors multiple protein-protein interactions that are required for proper sterol synthesis [6]. It is known that ergosterol synthesis is upregulated in the presence of azole antifungal drugs. This is due to the increased transcriptional activity of the Upc2 transcription factor [5]. Interestingly, mutations have been identified in *UPC2* in resistant clinical isolates [7]. Presently, the post-translational modifications regulating ergosterol synthesis are poorly understood. *in vitro* proteomic data suggest that several Erg proteins are ubiquitinated [8]. Whether ubiquitination promotes proteosome degradation has yet to be explored. There is a single report that has suggested there may be a relationship between the SLT2 mitogen activated kinase and sterol biosynthesis [9].

Azoles drugs block ergosterol biosynthesis in all yeast species [10]. The Erg11 lanosterol 14-α-demethylase enzymatic reaction is rate-limiting step in ergosterol biosynthesis. Erg11 itself is the direct target of the azole antifungal drugs [11]. It is highly conserved among pathogenic and non-pathogenic fungi [12]. It is well established that overexpressing *CaERG11* leads to azole resistance, as well as, mutations that alter Erg11 azole binding affinity [13–15]. Mutations in Erg11 that reduce azole binding affinity, and thus increase azole resistance, have been mapped and characterized. All mutations cluster around three regions of the CaErg11 protein: 105–165, 266–287, and 405–488 [16, 17]. Orthologous Erg11 azole resistance mutations have also been mapped in non-*albicans Candida spp*. [18].

**Fig 1:**
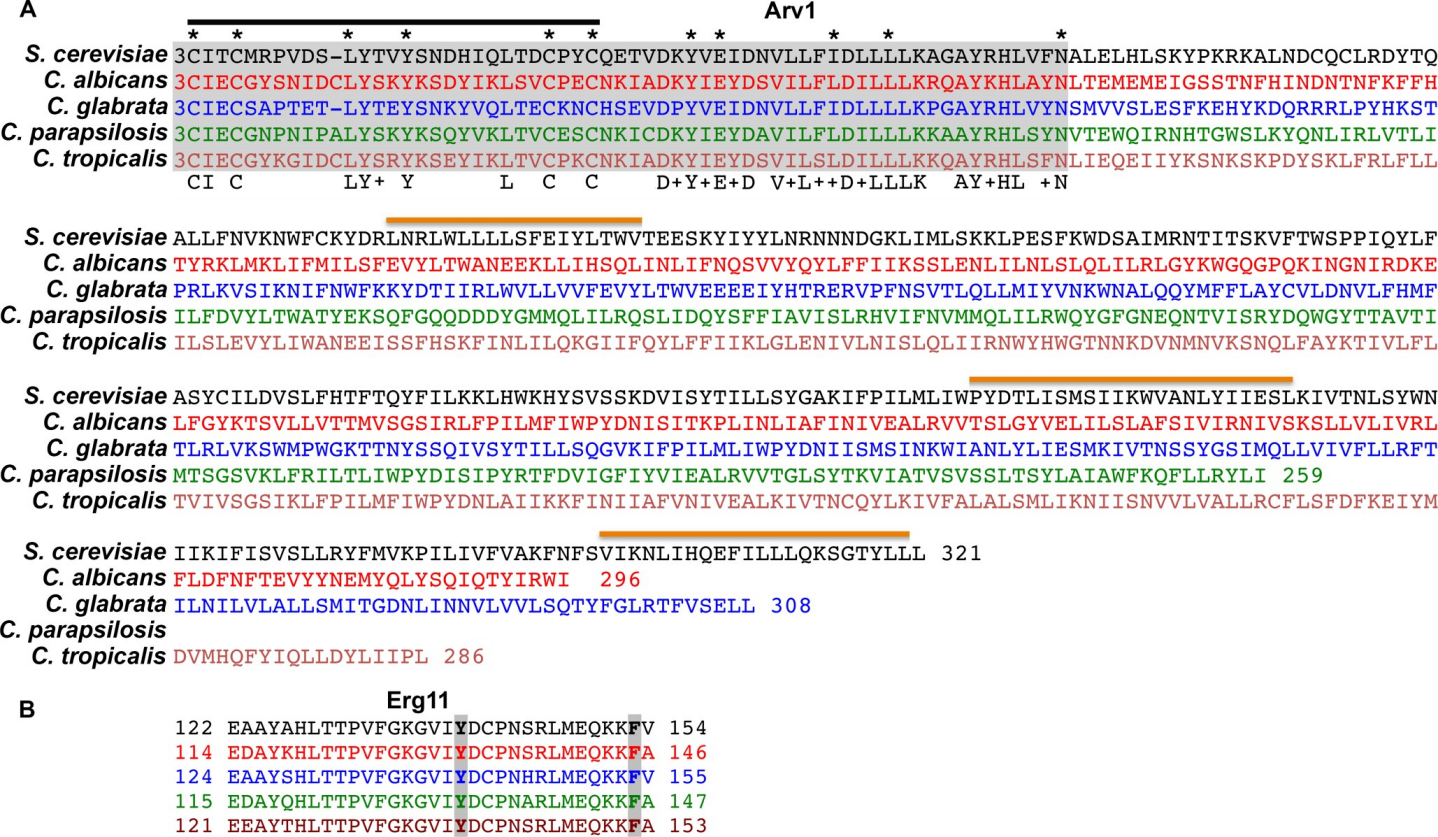
Amino acid sequence alignments of fungal Arv1 and Erg11 proteins. (A) Amino acid sequences of Arv1 proteins from *S*. *cerevisiae*, *C*. *albicans*, *C*. *glabrata*, *C*. *parapsilosis*, and *C*. *tropicalis*. Ahd (shaded gray); zinc-binding motif (black line); conserved amino acids (lower row); transmembrane domains (orange line); asterisks, (mutated amino acids). All sequences start at amino acid 3. (B) Fungal Erg11 amino acid sequences are shown containing conserved Tyr and Phe residues (shaded gray).

The loss of the *S*. *cerevisiae* Are1 and Are2 acyl-CoA:sterol acyltransferases responsible for the synthesis of ergosteryl esters results in accumulation of unesterified sterol, but cells remain viable [19–21], whereas the loss of *ARV1* (**a**re1 are2 **r**equired for **v**iability **1**) in the same background results in inviability [22]. Arv1 is a three transmembrane protein that contains an **A**rv1 **h**omology **d**omain (**Ahd**)^1^ that is highly conserved across fungal species (Fig 1A; Ahd, shaded gray) [23]. Within the AHD is a conserved putative zinc-binding motif that contains two CXXC motifs separated by ~20 amino acids (Fig 1A; black line) [23, 24].

The exact function of Arv1 has yet to be elucidated, but phenotypes that are associated with its loss suggest roles in regulating global lipid homeostasis. *Scarv1* cells are temperature sensitive and harbor pleiotrophic phenotypes that include azole hypersusceptibility [25], defects in sterol synthesis and localization that cause the accumulation of the Erg11 substrate lanosterol [26], and defects in phospholipid [26], sphingolipid [26], and glycosylphosphatidylinositol homeostasis [27]. It is also known that *Scarv1* cells have ER morphology defects and mislocalize proteins residing within this organelle [28], have an activated unfolded protein response (UPR) [29], and are hypersensitive to fatty acid supplementation [30].

Here, we used a structure/function approach to identify conserved amino acids within ScArv1 that were required for maintaining normal azole drug susceptibility. We uncovered several amino acid residues within the ScAhd. *Scarv1* cells expressing these specific mutant proteins were hypersusceptible to azole antifungals and had defects in sterol synthesis and localization. Mutant strains had reduced levels of ScErg11, most likely due to accelerated proteosomal degradation. Interestingly, we uncovered a physical interaction between ScArv1 and ScErg11 that was lost in azole hypersusceptible mutants. Identical results were recapitulated in *Scarv1* cells expressing orthologous CaArv1 mutants. Finally, we found that *Caarv1/Caarv1* strains expressing hypersusceptible CaArv1 mutants that lacked the ability to interact with CaErg11 lacked virulence. Overall our work suggests a direct correlation between Arv1 function and virulence. This may be due to it functioning in the maintenance of Erg11 stability. Thus, establishing a functional Erg11-Arv1 complex may be important for maintaining the virulence of *C*. *albicans*.

## Results

### Full-length Arv1 is required for maintaining normal antifungal susceptibility

Tinkelenberg *et al*., [22] showed that *Scarv1* cells were hypersusceptible to the polyene antifungal, nystatin. We previously showed that *Scarv1* cells were also hypersusceptible to azole antifungals [25]. In order to delineate what portions of ScArv1 were required for normal azole susceptibility, we ectopically expressed full-length Arv1-HA (Arv1-HA), Ahd-HA (Ahd-HA) alone, or an Arv1 protein lacking the Ahd (ΔAhd-HA) from the endogenous *ScARV1* promoter using a low copy centromeric plasmid. We then tested whether these truncations were able to confer normal antifungal susceptibility to *Scarv1* cells.

**Fig 2:**
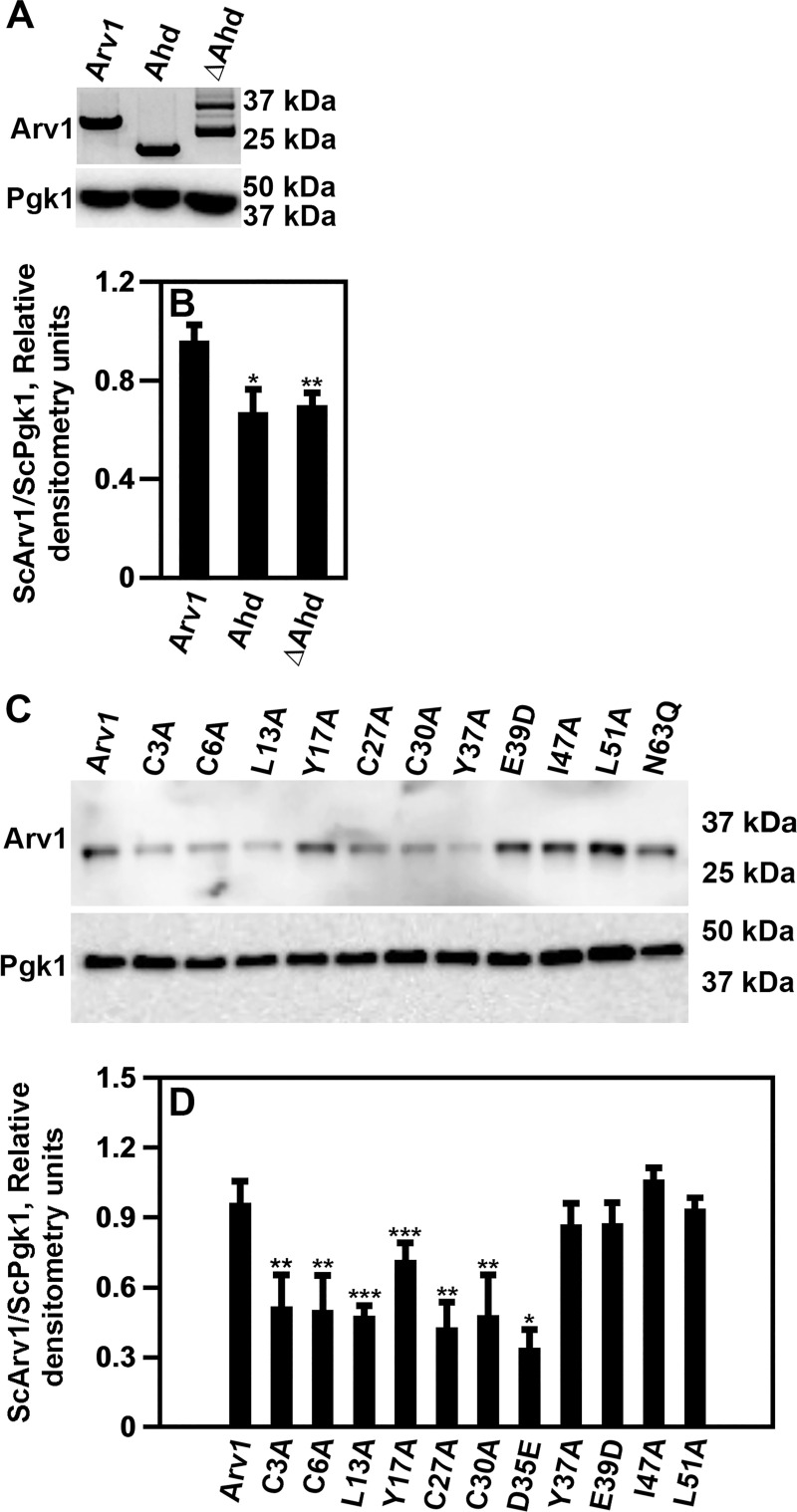
The expression of specific ScArv1 mutants result in reduced protein levels. Cells were grown to exponential phase (0.3–0.5 X 10^7^ cells/ml) and used to obtain a total cell lysate. Western analysis was used to determine the size and protein levels of ScARV1-HA mutants (anti-HA monoclonal antibody (clone 12CA5). ScPgk1 level was used as a loading control. (A) Protein levels of Arv1 truncations. (B) Relative protein expression of truncations. (C) Protein levels of ScArv1 point mutants. (D) Relative protein expression of point mutants. The westerns presented and densitometry values are representative of 5 individual experiments. Relative protein expression was determined using the densitometry values obtained for Arv1-HA proteins (numerator) compared to the ScPgk1 loading control (denominator). *p≤0.*01*; ***p≤0.*001*; ***p≤0.*0001*.

Our western analysis did indicate that the Ahd-HA and ΔAhd-HA proteins were expressed to lower levels then Arv1-HA (Fig 2A & 2B). It has been shown previously that *Scarv1* cells expressing the ΔAhd retain some Arv1 functions [31].

We used the antifungal drugs itraconazole, fluconazole, lovastatin, fenpropimorph, and terbinafine. A subset of these antifungals targets the Erg11 reaction in the ergosterol biosynthetic pathway (itraconazole and fluconazole), as well as reactions upstream (lovastatin) and downstream (fenpropimorph, and terbinafine) of Erg11.

We found that S*carv1* cells were hypersusceptible to itraconazole and fluconazole azole antifungal drugs (Table 1). These cells were also hypersusceptible to terbinafine and fenpropimorph, while showing normal susceptibility to lovastatin (Table 1). *Scarv1* cells expressing ScAhd or ScΔAhd had similar susceptibility profiles. Wild type and *Scarv1* cells expressing full-length ScArv1 showed normal susceptibility to all antifungal drugs tested. Thus, neither the ScAhd nor ScΔAhd could replace full-length ScArv1 and restore normal antifungal susceptibility to *Scarv1* cells treated with several antifungal drugs, which target different steps in the ergosterol biosynthetic pathway. We point out that normal susceptibility to lovastatin seems reasonable, as this antifungal drug targets HMG-CoA reductase, which is an enzyme upstream of the Erg11 enzymatic step.

**Table 1:** Antifungal susceptibilities of *Scarv1* site-directed mutant strains.

Strain	Itraconazole	Fluconazole	Terbinafine	Fenpropimorph	Lovastatin
*W303****a*** *(WT)*	0.24 ± 0.03	0.47 ± 0.02	6.9 ± 0.38	4.3 ± 0.83	39 ± 2.6
*arv1*	0.01 ± 0.01**	0.12 ± 0.03**	0.38 ± 0.04***	0.72 ± 0.13***	31 ± 1.3
*arv1 + ARV1*	0.25 ± 0.06	0.48 ± 0.04	9.6 ± 1.2	5.7 ± 0.89	43 ± 1.5
*arv1 + Ahd*	0.09 ± 0.03**	0.08 ± 0.04**	0.14± 0.05***	1.1 ± 0.08***	41 ± 2.3
*arv1 + ΔAhd*	0.13 ± 0.05**	0.07 ± 0.03**	1.6 ± 0.6***	1.5 ± 0.21***	36 ± 2.4
*arv1 + ARV1*^*C3A*^	0.26 ± 0.03	0.60 ± 0.05	5.8 ± 0.8	6.1 ± 1.3	51 ± 3.7
*arv1 + ARV1*^*C6A*^	0.33 ± 0.08	0.62 ± 0.05	7.9 ± 2.7	3.9 ± 0.66	33 ± 2.5
*arv1 + ARV1*^*L13A*^	0.02 ± 0.00**	0.13 ± 0.02***	0.76± 0.33***	0.15 ± 0.03***	27 ± 3.8
*arv1 + ARV1*^*Y17A*^	0.17 ± 0.04	0.53 ± 0.03	7.3 ± 0.41	5.5 ± 0.42	29 ± 1.4
*arv1 + ARV1*^*C27A*^	0.06 ± 0.03**	0.15 ± 0.03**	1.8 ± 0.24***	0.95 ± 0.01***	36 ± 1.7
*arv1 + ARV1*^*C30A*^	0.25 ± 0.02	0.63 ± 0.07	6.4 ± 1.4	4.8 ± 1.1	44 ± 3.6
*arv1 + ARV1*^*Y37A*^	0.19 ± 0.01	0.58 ± 0.04	8.4 ± 2.1	6.1 ± 0.26	36 ± 2.4
*arv1 + ARV1*^*E39D*^	0.17 ± 0.04	0.44 ± 0.03	6.1 ± 0.34	4.4 ± 0.81	41 ± 4.1
*arv1 + ARV1*^*I47A*^	0.05 ± 0.04*	0.09 ± 0.01**	0.49 ± 0.19***	1.4 ± 0.04***	46 ± 6.2
*arv1 + ARV1*^*L51A*^	0.21 ± 0.04	0.72 ± 0.07	6.7 ± 0.71	3.9 ± 0.12	48 ± 3.7
*arv1 + ARV1*^*N63Q*^	0.10 ± 0.02**	0.41 ± 0.01**	0.84 ± 0.03***	3.1 ± 0.03***	51 ± 7.6

^a^values are μg/ml.

^b^*P<0.01

**P<0.001

***P<0.0001.

### Conserved amino acids within the Ahd are required for maintaining normal antifungal susceptibility

The amino acid sequence alignments of Arv1 proteins from *S*. *cerevisiae*, *C*. *albicans*, *C*. *glabrata*, *C*. *parapsilosis*, and *C*. *tropicalis* are shown (Fig 1A). 25 conserved amino acid residues were found within the Ahd’s of these species (bottom row). No homology was found outside the ScAhd. Thus, we focused on identifying conserved amino acids within the ScAhd that were required for normal antifungal susceptibility.

We used site-directed mutagenesis and mutated a subset of these conserved amino acids (L13A, Y17A, Y37A, E39D, I47A, L51A, and N63Q) (Fig 1A; asterisk). We also mutated the conserved cysteine residues Cys3 and Cys6, and Cys27 and Cys30 that are found within the zinc-binding domain to alanines (Fig 1A; asterisk). We ectopically expressed individual full-length Arv1-HA mutants in *Scarv1* cells using a low copy centromeric plasmid, and subsequently determined their growth in the presence of antifungal drugs.

Arv1 mutant protein levels are shown in Fig 2C. A number of strains had reduced detectable levels of Arv1 protein (Fig 2C & 2D). However, while a number of these mutants were expressed at low levels, they were still able to confer normal antifungal susceptibility to *Scarv1* cells (*see below*).

We found that *Scarv1* cells expressing the ScArv1^L13A^, ScArv1^C27A^, ScArv1^I47A^, or ScArv1^N63Q^ mutants were hypersusceptible to itraconazole, fluconazole, terbinafine, fenpropimorph, but had normal susceptibility to lovastatin (Table 1). *Scarv1* cells expressing all other ScArv1 mutants had normal susceptibility to all antifungal drugs tested (ScArv1^C3A^, ScArv1^C6A^, ScArv1^Y17A^, ScArv1^C30A^, ScArv1^Y37A^, ScArv1^E39D^, and ScArv1^L51A^).

Further examination of the protein levels and the azole hypersusceptibility of various mutants revealed that *Scarv1* cells expressing ScArv1^C27A^ remained azole hypersusceptible, but had a protein level that was similar to that detected for ScArv1^Y37A^, whose expression was able to confer normal azole susceptibility. Moreover, while ScArv1^Y17A^ and ScArv1^YI47A^ were expressed to similar levels, only cells expressing ScArv1^Y17A^ showed normal susceptibility. Thus, there does not seem to be a direct correlation between reduced detectable ScArv1 protein and azole hypersusceptibility.

Possible explanations for these results may be that lower expressing mutants, which retain activity, localize to a membrane fraction that is resistant to extraction, or may be more tightly associated with the endoplasmic reticulum, again making it difficult to extract. We did not examine if any protein was associated with detergent resistant membranes/rafts [32], or were more tightly retained in the ER [26, 28].

### *Scarv1* cells expressing the ScArv1^C27A^ mutant have altered sterol composition

We previously showed that *Scarv1* cells accumulated lanosterol and unknown sterol intermediates, suggesting a role for ScArv1 in sterol synthesis [26]. Based on these results, we determined the sterol compositions for a subset of mutants using gas chromatography/mass spectrometry. We chose the mutants to be analyzed based on whether they did or did not suppress azole hypersusceptibility, and protein expression levels. ScArv1^C3A^ and ScArv1^Y37A^ were chosen as mutants able to restore normal antifungal susceptibility to *Scarv1* cells, while ScArv1^C27A^ was chosen as an antifungal hypersusceptible mutant.

**Fig 3:**
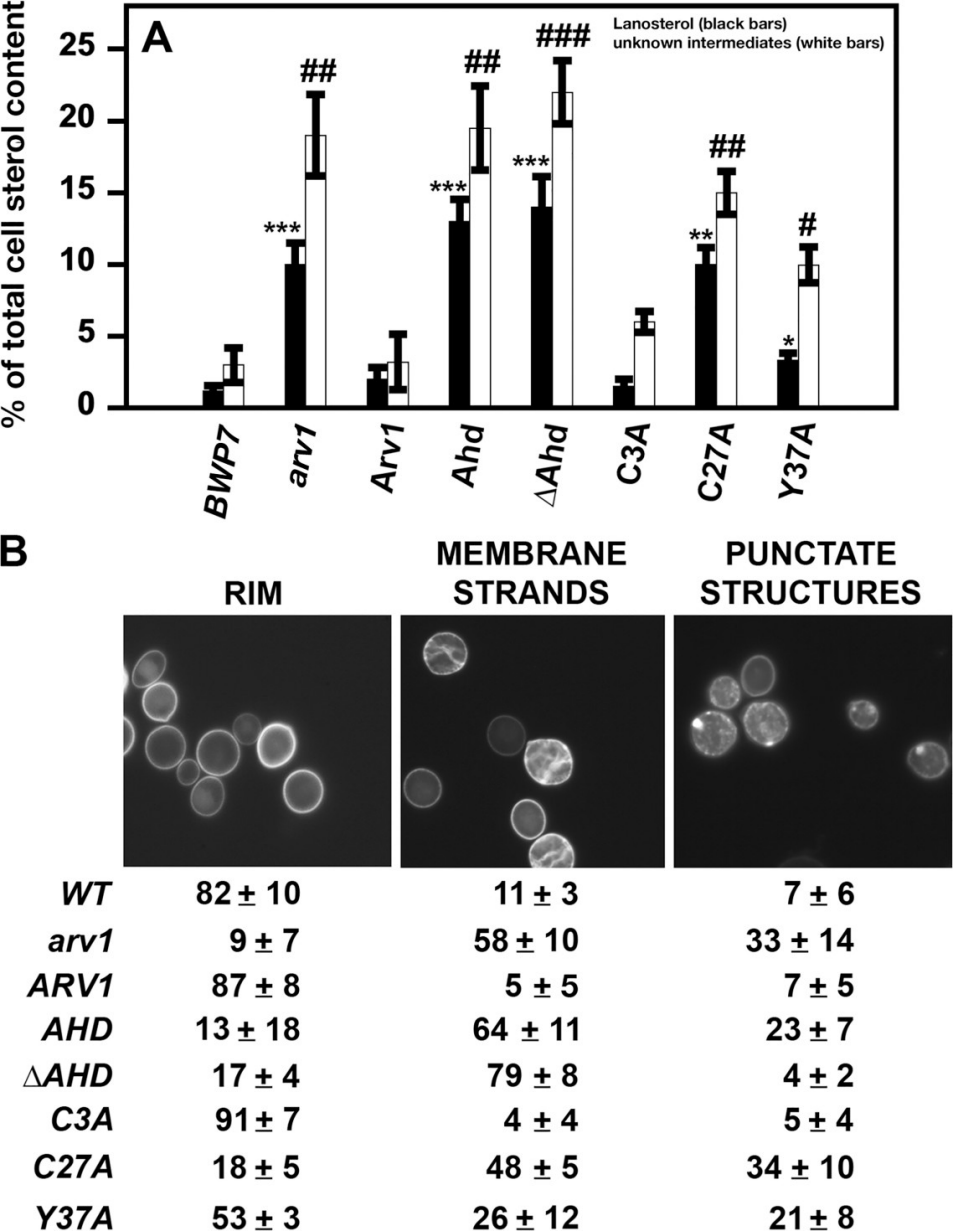
*Scarv1* expressing ScArv1^C27A^ and ScArv1^Y37A^ are unable to maintain proper sterol composition and localization. (A) Sterol composition and localization was determined in *Scarv1* cells expressing ScArv1 mutants. Sterol composition was determined by LC/MS as described in Materials and Methods. Sterol levels were plotted as a percentage of total cellular sterol content. Data represent the percentages of lanosterol (*black bars*) and unknown sterol intermediates (*white bars*). (B) The localization of unesterified sterol was visualized using filipin staining and fluorescence microscopy. Values are the percentage of cells with the indicated structures. The data are representative of 5 individual double-blinded experiments. 300–500 cells were analyzed for each individual experiment. *p≤0.*01*; **p≤0.*001*; ***p≤0.*0001*; ## *p≤0*.*01*; ## *p≤0*.*001*; ### *p≤0*.*0001*.

We found that *Scarv1* cells expressing Arv1 had a normal sterol composition, whereas the sterol composition was altered in *Scarv1* cells expressing the ScAhd or ScΔAhd truncations. These cells accumulated lanosterol (*) and unknown sterol intermediates (#) (Fig 3A) [26]. Moreover, *Scarv1* cells expressing ScArv1^C27A^ or ScArv1^Y37A^ also accumulated lanosterol and unknown sterol intermediates (Fig 3A).

### *Scarv1* cells expressing ScArv1^C27A^ have altered sterol localization

Our studies [23, 25], as well as those from the Beh and Mennon laboratories [28], showed that *Scarv1* cells were unable to properly localize sterols. Thus, we visualized sterol localization in *Scarv1* cells expressing mutant proteins using filipin staining. Sterol localization was visualized by fluorescence microscopy. 300–500 cells were analyzed, and the percentages of specific sterol structures were determined.

We found that exponentially growing *Scarv1* cells expressing ScArv1 had the majority of their sterol localized to the plasma membrane rim (Fig 3B. *RIM*; *ARV1*), while *Scarv1* cells contained several aberrant types of sterol-containing structures, including membrane strands, which may be sterol-accumulating organelle membranes, and punctate structures that are mainly associated with the plasma membrane [28]. These structures were still seen at stationary phase of growth, suggesting that there was not a slower sterol localization defect in these cells.

While *Scarv1* cells expressing ScArv1^C3A^ also showed rim staining, *Scarv1* cells expressing ScArv1^C27A^ or ScArv1^Y37A^ harbored sterol localization defects that were similar to those seen for *Scarv1* cells (Fig 3B). These cells accumulated both intercellular membrane strands and plasma membrane-associated punctate structures to high degrees (Fig 3B).

### Erg11 protein levels are reduced in *Scarv1* cells expressing ScArv1^C27A^

ScErg11 is the direct target of azoles. We previously showed a direct correlation between azole hypersusceptibility and reduced steady-state levels of ScErg11 in *Scarv1* cells [25]. Normal ScErg11 levels could be restored to *Scarv1* cells if ScArv1 or CaArv1 was expressed [25], again demonstrating a high degree of conservation. To further delve into the mechanism(s) responsible for the azole hypersusceptibility and sterol defects of *Scarv1* cells, we determined ScErg11 levels in *Scarv1* cells expressing mutant proteins.

**Fig 4:**
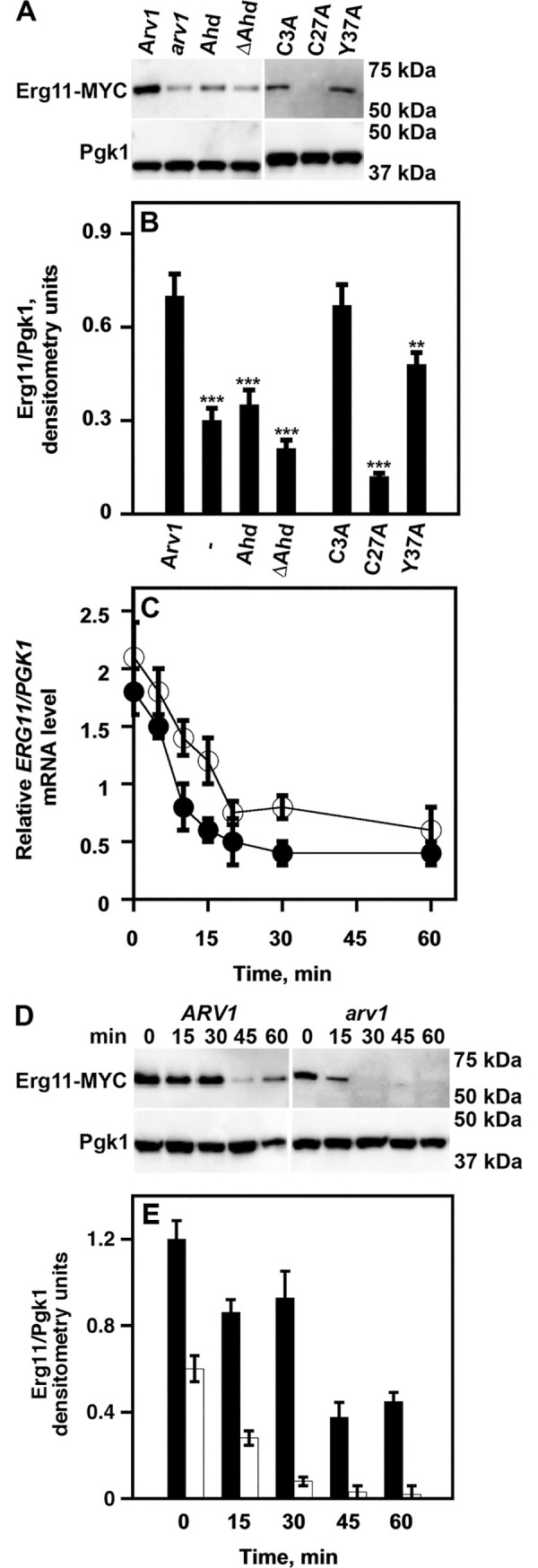
*Scarv1* expressing ScArv1^C27A^ and ScArv1^Y37A^ are unable to maintain normal steady-state levels of ScErg11. Whole cell lysates from *Scarv1* cells expressing ScArv1 mutants were resolved by SDS-PAGE and transferred to nitrocellulose. ScErg11-MYC levels were determined by western analysis using an anti-MYC monoclonal antibody (9E10). ScPgk1 was used as a loading control. (A) ScErg11-MYC levels in *Scarv1* cells expressing Arv1 mutants. (B) Relative protein expression of ScErg11-MYC compared to ScPgk1. Relative protein expression was determined using the densitometry values obtained for ScErg11-MYC protein (numerator) compared to the ScPgk1 loading control (denominator). The data shown are representative of 5 individual experiments. (C) *ScERG11* and *ScPKG1* mRNA expression levels. Thiolutin, which is an inhibitor of bacterial and yeast RNA polymerases, was used at 3μg/ml. The data presented are representative of 5 individual experiments. *WT*, closed circles; *arv1*, open circles. (D) The half-life of ScErg11-MYC was determined by pulse-chase kinetic experiments where expression was driven from the *GAL1* promoter and initiated by addition of galactose. The indicated times are post addition of glucose, which stops *GAL1*-driven protein expression. Protein lysates were obtained and protein level was determined using western analysis and an anti-MYC monoclonal antibody (9E10). (E) Relative densitometry units are representative of the data shown in panel D. *WT*, black bars; *arv1*, white bars. The data are representative of 5 individual experiments. **p≤0.*001*; ***p≤0.*0001*.

Wild type levels of ScErg11-MYC were seen in *Scarv1* cells expressing full-length ScArv1, whereas levels were reduced in *Scarv1* cells, and *Scarv1* cells expressing ScAhd or ScΔAhd alone (Fig 4A & 4B). *Scarv1* cells expressing ScArv1^C3A^, ScArv1^C27A^ or ScArv1^Y37A^ also had reduced levels of ScErg11-MYC.

### Erg11 instability in *Scarv1* cells expressing ScArv1^C27A^ is due to accelerated protein degradation

We wanted to understand what mechanism was responsible for reducing the steady state level of Erg11. We first determined whether *ScERG11* mRNA was unstable. The ½ life of *ScERG11* mRNA was compared to the ½ life of *ScPGK1* mRNA. We found no differences in mRNA stability between *ScERG11* (*open circles*) and *ScPGK1* (*closed circles*) (Fig 4C). Similar results were seen in *Scarv1* cells expressing Ahd, ΔAhd, ScArv1^C3A^, ScArv1^C27A^, or ScArv1^Y37A^ (not shown). Thus, the reduced ScErg11-MYC protein level seen in *Scarv1* cells expressing Scarv1^C27A^ was not due to the accelerated decay of *ScERG11* mRNA.

We next analyzed whether Erg11 was being degraded faster. Thus, we determined its ½ life in *Scarv1* cells. We did this by following the synthesis and degradation of a ScErg11-MYC protein, whose expression was under the control of the inducible *GAL1* promoter. Cells were grown in 2% raffinose to exponential phase and shifted to galactose for 2 hr to induce protein expression. After 2hr, glucose was added to shut off galactose-inducible protein expression, and ScErg11 levels were determined at the indicated times post glucose addition using western analysis. Cyclohexamide (CHX) was added during glucose addition in order to inhibit protein translation.

We found that ScErg11 ½ life was ~45 min in *Scarv1* cells expressing ScArv1, while it was ~15 min in *Scarv1* cells (Fig 4D & 4E). The densitometry units shown in Fig 4E are representative of the levels of ScErg11-MYC in Fig 4D.

We next examined if the reduced ½ life of ScErg11 in *Scarv1* cells was due to accelerated proteosomal degradation. We chose to look at ScErg11 levels at 45 min, the ½ life of ScErg11-MYC in *ScARV1* cells, as this time point gave us the most consistent results. Pulse-chase experiments were again used, and cells were grown in the absence or presence of the proteosome inhibitor, MG132.

**Fig 5:**
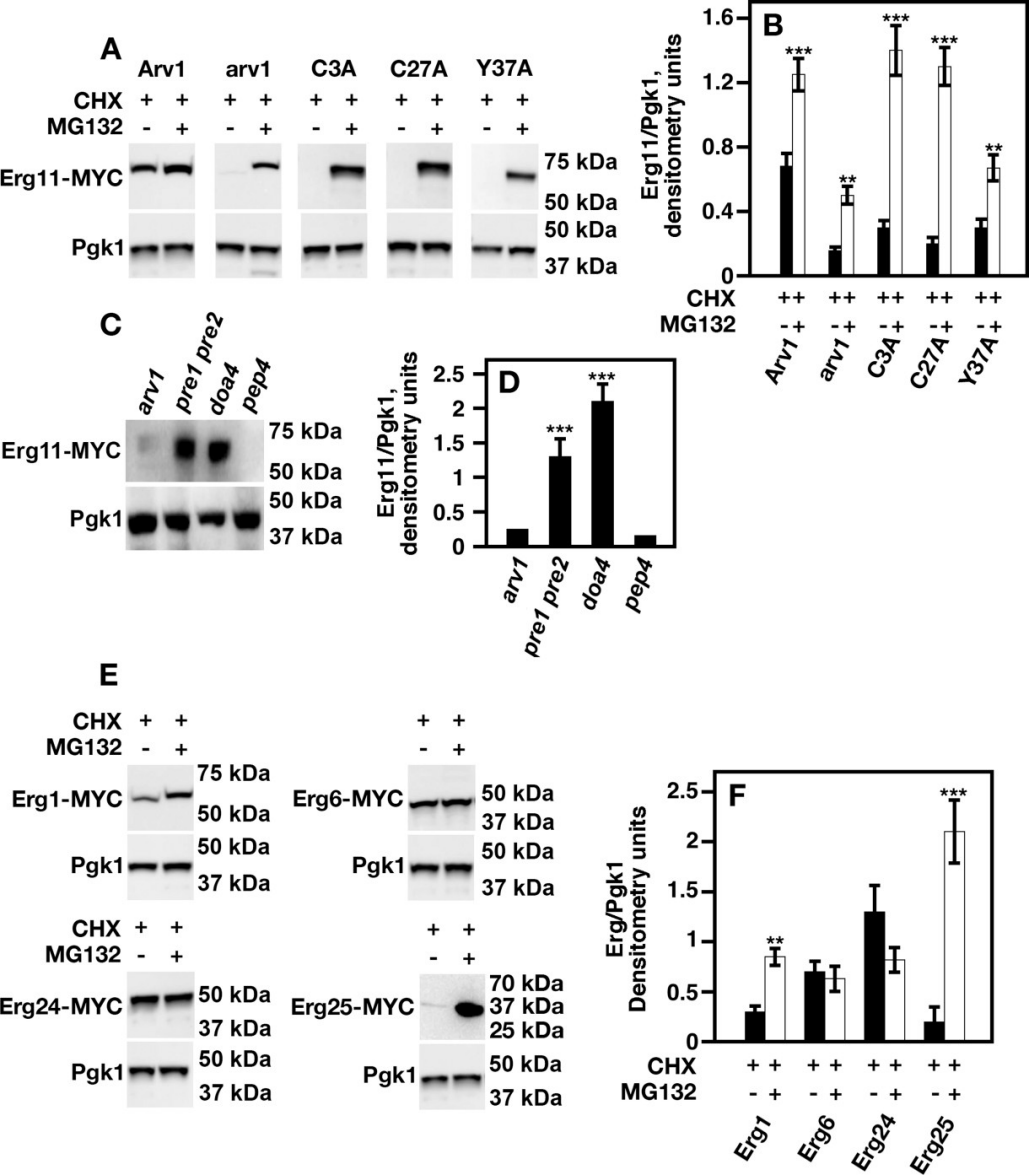
ScErg11 protein level is stabilized in Scarv1 cells expressing ScArv1^C27A^ in the presence of the proteosome inhibitor, MG132. Cells were treated with cyclohexamide (translation inhibitor) in the absence or presence of MG132 (proteosome inhibitor) for 45 min. ScErg1/6/11/24/25-MYC levels were determined by western analysis using an anti-MYC monoclonal antibody (9E10). ScPgk1 was used as a loading control. (A) ScErg11 levels were determined in cyclohexamide-treated *Scarv1* cells expressing Arv1 mutants in the absence and presence of MG132 for 45 min. (B) Relative densitometry units are representative of the data shown in panel A. (C) ScErg11 levels were determined in cells lacking proteosomal chymotrypsin-like activity (*pre1 pre2*), ubiquitin-dependent degradation (*doa4*), or carboxypeptidase activity (*pep4*). (D) Relative densitometry units are representative of the data shown in panel C. (E) ScErg1/6/24/25-MYC levels were determined in cyclohexamide-treated *Scarv1* cells in the absence and presence of MG132 for 45 min. (F) Relative densitometry units are representative of the data shown in panel E.

The addition of MG132 to *Scarv1* cells expressing ScArv1 stabilized ScErg11-MYC when compared to cells grown in the absence of MG132 (Fig 5A; *CHX vs*. *CHX + MG132*). MG132 addition also stabilized ScErg11-MYC in *Scarv1* cells, albeit not to the same level observed for *Scarv1* cells expressing ScArv1 (Fig 5A; Arv1 *vs*. arv1, *CHX vs*. *CHX + MG132*). While *Scarv1* cells expressing ScArv1^C3A^, ScArv1^C27A^, or ScArv1^Y37A^, had lower levels of Erg11-MYC in the absence of MG132 (Fig 5A; *CHX*), protein levels became stable in these cells in the presence of MG132 (Fig 5A; *CHX vs*. *CHX + MG132*). These results suggested that accelerated proteosome degradation might play a role in causing Erg11-MYC instability in *Scarv1* cells expressing ScArv1^C27A^. The densitometry units shown in Fig 5B are representative of the levels of ScErg11-MYC in Fig 5A.

To determine if accelerated proteosome degradation was solely responsible for Erg11-MYC instability, we analyzed Erg11-MYC levels in *Scarv1* cells lacking proteosome chymotrypsin-like activity (*pre1-1 pre2-2*), deubiquitination recycling activity (*doa4*), or carboxypeptidase activity (*pep4*). Interestingly, Erg11-MYC steady state levels were stable in *Scarv1* cells lacking Pre1/Pre2 or Doa4 activity, but remained unstable in cells lacking Pep4, when compared to *Scarv1* cells (Fig 5C). The densitometry units shown in Fig 5C are representative of the levels of ScErg11-MYC in Fig 5B.

Finally, we determined whether the stability of additional Erg proteins was dependent on the presence of Arv1. We generated *Scarv1* cells expressing ScErg1-MYC, ScErg6-MYC, ScErg24-MYC, or ScErg25-MYC, and tested protein stability in the absence or presence of MG132 at 45 min. Both ScErg1-MYC and ScErg25-MYC were expressed at low levels in *Scarv1* cells, but protein levels became stable in the presence of MG132 (Fig 5E; *CHX vs*. *CHX + MG132*). Interestingly, both ScErg6-MYC and ScErg24-MYC were expressed at the same level either in the absence or presence of MG132 (Fig 5E; *CHX vs*. *CHX + MG132*). The densitometry units shown in Fig 5F are representative of the levels of ScErg1-MYC, ScErg6-MYC, ScErg24-MYC, and ScErg25-MYC in Fig 5E. Thus, the presence of ScArv1 seems to be necessary for maintaining the stability of a subset of enzymes required for ergosterol biosynthesis.

### Erg11 physically interacts with Arv1

Johnsson and Varshavsky [33] first described the split-ubiquitin two-hybrid system for looking at the interactions between membrane-bound proteins. This method takes advantage of the N-terminal (Nub) and C-terminal (Cub) halves of ubiquitin to detect protein-protein interactions. The Nub and Cub protein fragments are fused to bait and prey proteins, respectively. The Cub protein itself is a chimera that contains a transcription factor (protein-A-LexA-VP16) that is released upon protein-protein binding, whereby it can activate reporter gene expression. Expression of the *HIS3* gene allows for growth in the absence of histidine and β-galactosidase activity from the *LacZ* gene.

Bard and colleagues previously demonstrated that ScArv1 physically interacted with ScErg11 using the split-ubiquitin two-hybrid system [6]. They also demonstrated several ScErg protein-protein interactions that were necessary for sterol synthesis [34]. Moreover, they found that the level of the C-24 sterol methyltransferase Erg6 was decreased in *erg28* cells. Erg28 acts as a scaffold, nucleating the ergosome that is responsible for sterol biosynthesis [35]. Based on these results, we tested whether ScErg11 stability was dependent on its ability to interact with ScArv1, and ScArv1 truncations or mutants. Positive and negative controls were designed based on the strong interaction between ScErg28 and Erg27, and the lack of interaction between ScErg28 and ScErg9, respectively [6].

**Fig 6:**
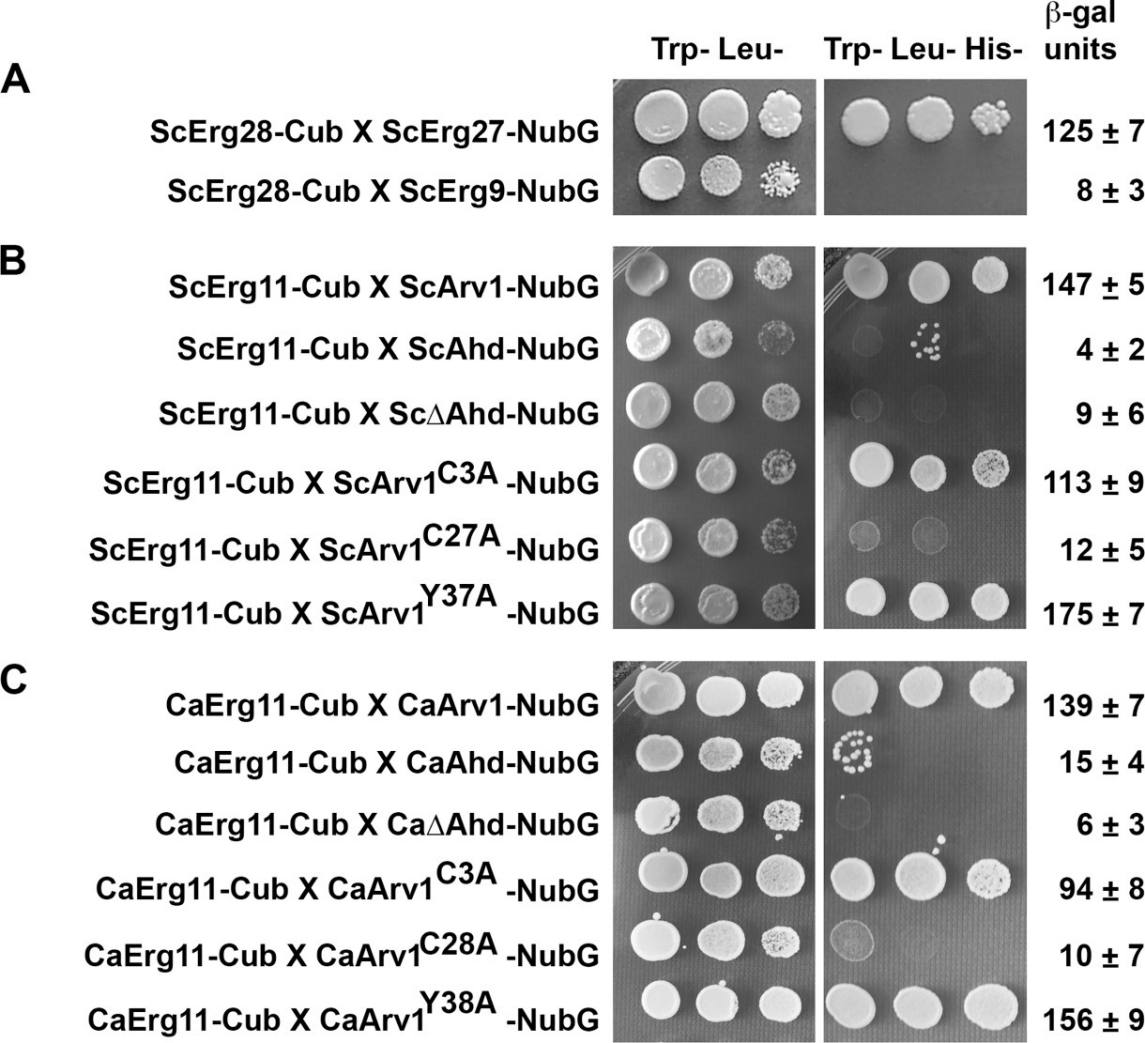
Both ScArv1^C27A^ and CaArv1^C28A^ are unable to interact with ScErg11 and CaErg11, respectively, using the split-Ub two-hybrid system. Cells expressing chimeric forms of ScArv1/CaArv1 were grown to exponential phase in the appropriate plasmid selection medium. Serial dilutions of cells were dropped onto plates using a pin tool, and cell growth was visualized after 48 hr. True protein-protein interactions were deemed true if cells grew on Trp^-^Leu^-^His^-^ agar plates, and had a >3-fold increase in β-galactosidase activity over baseline. (A) Growth rates of ScErg28-Cub X ScErg9-NubG and ScErg28-Cub X ScErg27-NubG negative and positive interaction control cells, respectively. (B) Growth rates of cells expressing mutant forms of ScArv1 and full-length ScErg11. (C) Growth rates of cells expressing mutant forms of CaArv1 and full-length CaErg11. Data are representative of 5 individual experiments.

As previously reported, we detected an interaction between ScErg28 and ScErg27, as these cells grew in the absence of tryptophan, leucine, and histidine (Trp^-^ Leu^-^ His^-^), and had a 15-fold increase in β-galactosidase activity over that observed for the negative interaction control (ScErg28-Cub X ScErg9-NubG) (Fig 6A).

We next looked for an interaction between ScErg11 and full-length or truncated forms of ScArv1. We observed an interaction for ScErg11 and full-length ScArv1, but not between ScErg11 and the ScAhd or ScΔAhd fragments (Fig 6B). Interestingly, we also observed an interaction between ScErg11 and ScArv1^C3A^ and ScArv1^Y37A^, but not with ScArv1^C27A^ (Fig 6B). We next decided to examine whether these same interactions were conserved in *C*. *albicans*. We obtained the same results. CaErg11 interacted with full-length CaArv1, CaArv1^C3A^, and CaArv1^Y38A^, but did not interact with CaAhd, CaΔAhd, or CaArv1^C28A^ (Fig 6C).

**Fig 7:**
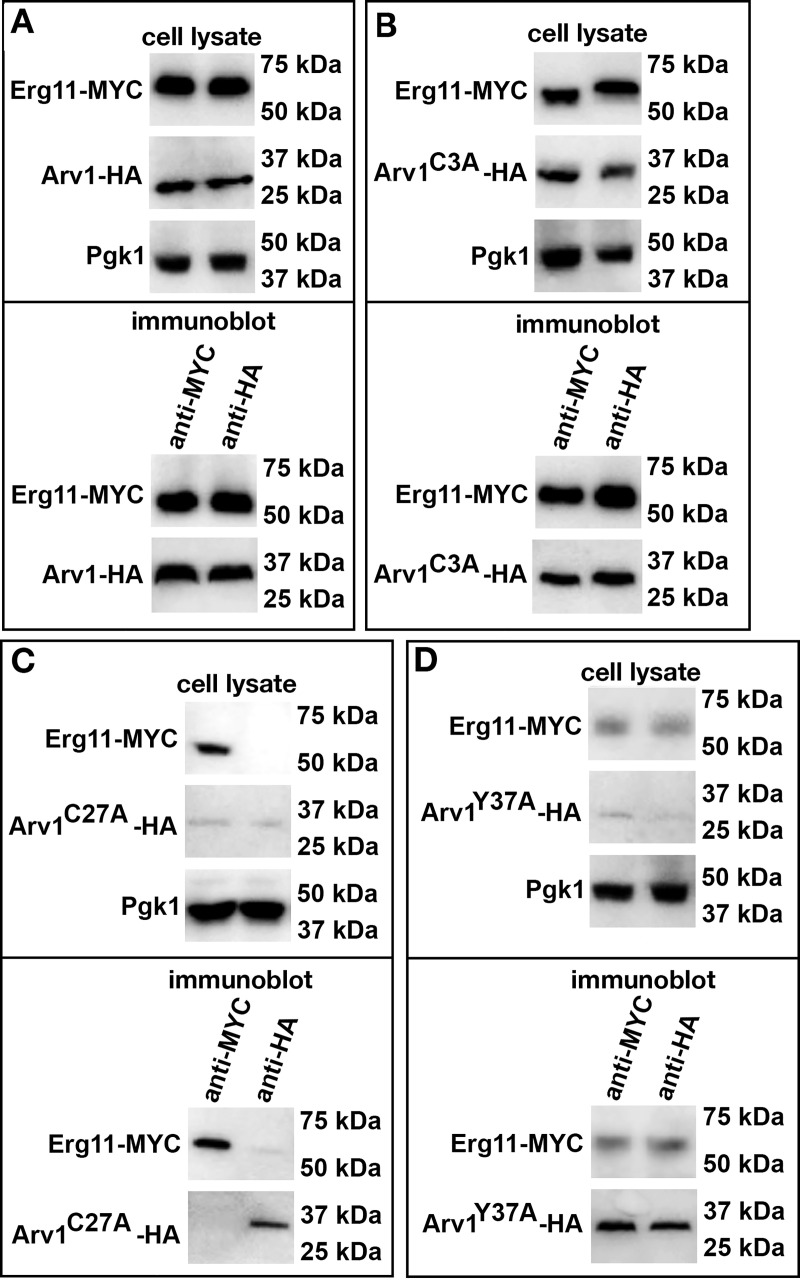
ScArv1^C27A^ does not co-immunoprecipitate with ScErg11. (A-D) *Scarv1* cells co-expressing wild type and mutant forms of ScArv1-HA and wild type ScErg11-MYC were grown to exponential phase in the appropriate drop out medium for plasmid selection. Whole cell lysates were used for co-immunoprecipitation experiments. ScArv1-HA and ScErg11-MYC levels were determined using western analysis using anti-HA monoclonal (12CA5) and monoclonal anti-MYC (9E10) antibodies, respectively. Pgk1 was used as a loading control. (A-D, immunoblot), represents the co-immunoprecipitation western results using the indicated anti-MYC antibodies or anti-HA antibodies and indicated Arv1 mutants. The cell lysate panels represent Erg11-MYC or Arv1-HA levels in total cell lysates. The difference in size of Erg11 in panel B may be due to post-translational modification. We have previously found that Erg11 migrates as a higher molecular weight species in certain Arv1 mutants (not shown). The differences in the detection observed for certain Arv1 mutants and Erg11 may be due to the use of protein lysates from steady state cells, and is strain dependent. The panels represent data from 3 individual experiments.

Next, we determined if we could recapitulate our two-hybrid results using co-immunoprecipitation experiments. The top panels of Fig 7A–7D (cell lysate) are westerns showing the total amount of each protein in cell lysates. The bottom panels of Fig 7A–7D are the co-immunoprecipitation results using western analysis (immunoblot).

We first examined whether ScErg11-MYC and ScArv1-HA could co-immunoprecipitate in *Scarv1* cells. We were able to pull down ScErg11-MYC with ScArv1-HA using either anti-MYC or anti-HA monoclonal antibodies (Fig 7A, immunoblot). We next co-expressed ScArv1^C3A^-HA, ScArv1^C27A^-HA, or ScArv1^Y37A^-HA, with ScErg11-MYC, and performed co-immunoprecipitation experiments. *Scarv1* cells expressing ScArv1^C3A^-HA had detectable levels of ScArv1^C3A^-HA and ScErg11-MYC (Fig 7A *vs*. 7B; cell lysate). We were able to co-immunoprecipitate ScErg11-MYC and ScArv1^C3A^-HA in these cells (immunoblot).

*Scarv1* cells expressing Arv1^C27A^-HA or ScArv1^Y37A^-HA had reduced levels of ScArv1-HA and ScErg11-MYC (Fig 7C *vs*. 7A; cell lysate). Importantly, we found that we were able to co-immunoprecipitate ScArv1^Y37A^-HA and ScErg11-MYC in *Scarv1* cells (Fig 7C, immunoblot), but were not able to co-immunoprecipitate ScArv1^C27A^-HA and ScErg11-MYC (Fig 7D, immunoblot).

### CaArv1 interacts with CaErg11 in *Scarv1* cells

**Fig 8:**
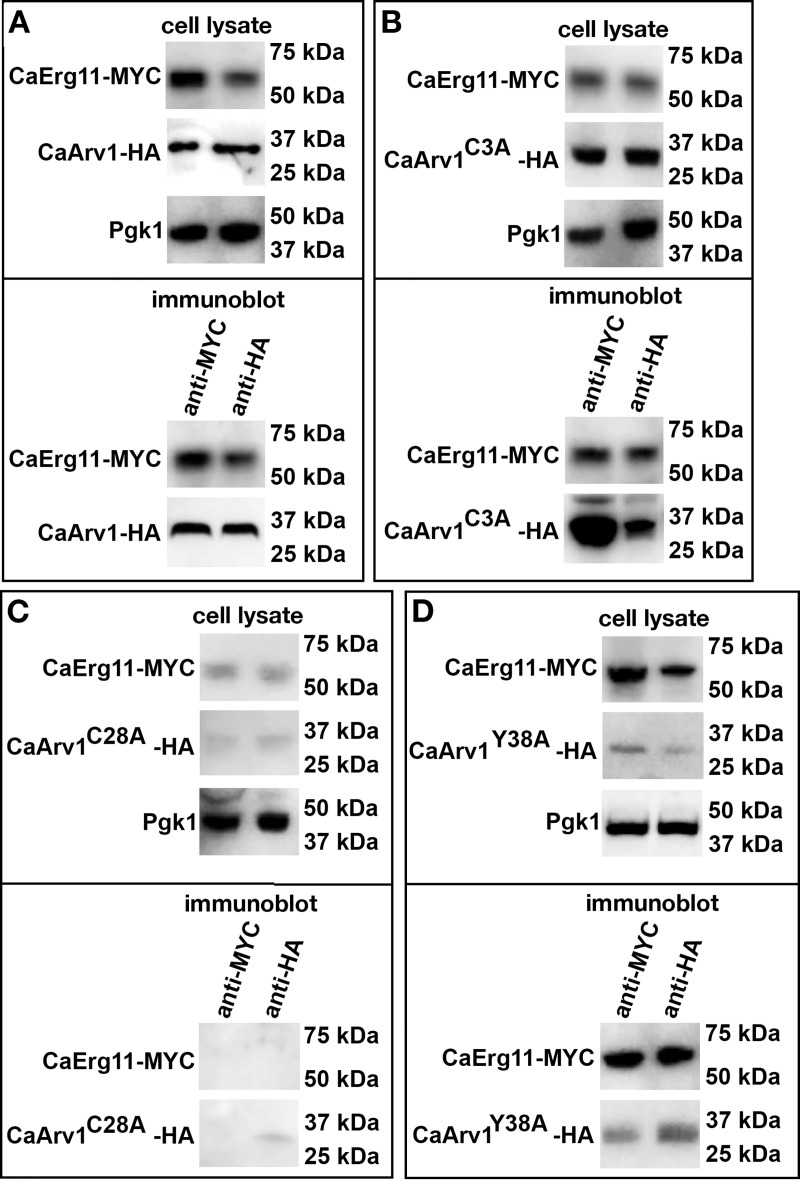
CaArv1^C28A^ is unable to co-immunoprecipitate with ScErg11. *Scarv1* cells co-expressing mutant CaArv1-HA and wild type CaErg11-MYC were grown to exponential phase in the appropriate plasmid selection medium. (A-D), represent the co-immunoprecipitation western results of *Scarv1* cells co-expressing CaArv1 mutants and CaErg11-MYC (immunoblot). The cell lysate panels represent Erg11-MYC or Arv1-HA protein levels in total cell lysates. The differences in the detection observed for certain Arv1 mutants and Erg11 may be due to the use of protein lysates from steady state cells, and is strain dependent. The panels represent data from 3 individual experiments.

We next tested if the physical interaction between ScArv1-HA and ScErg11-MYC was conserved in *C*. *albicans*. Here we expressed CaArv1^C3A^, and conserved CaArv1^C28A^ and CaArv1Y^38A^, in *Scarv1* cells expressing CaErg11-MYC. We observed the same co-immunoprecipitation results that we saw with the *S*. *cerevisiae* proteins. *Scarv1* cells expressing CaArv1-HA or CaArv1^C3A^-HA had normal levels of protein (Fig 8A & 8B, cell lysate), and each mutant was able to co-immunoprecipitate with CaErg11-MYC (Fig 8A & 8B). *Scarv1* cells expressing CaArv1^C28A^-HA or CaArv1^Y38A^ had reduced levels of CaArv1 protein (Fig 8C & 8D, cell lysate). However, we found that CaArv1^Y38A^-HA was able to co-immunoprecipitate with CaErg11-MYC, whereas CaArv1^C28A^-HA could not (Fig 8C *vs*. 8D, immunoblot).

### CaArv1^C28A^ does not interact with azole-resistant CaErg11^Y132L F145L^ in *Scarv1* cells

Many azole resistant clinical isolates express CaERG11^Y132L F145L^, which harbors altered azole-binding affinity (Fig 1B). We were interested in exploring whether CaArv1 had any role in stabilizing this clinically relevant protein.

**Fig 9:**
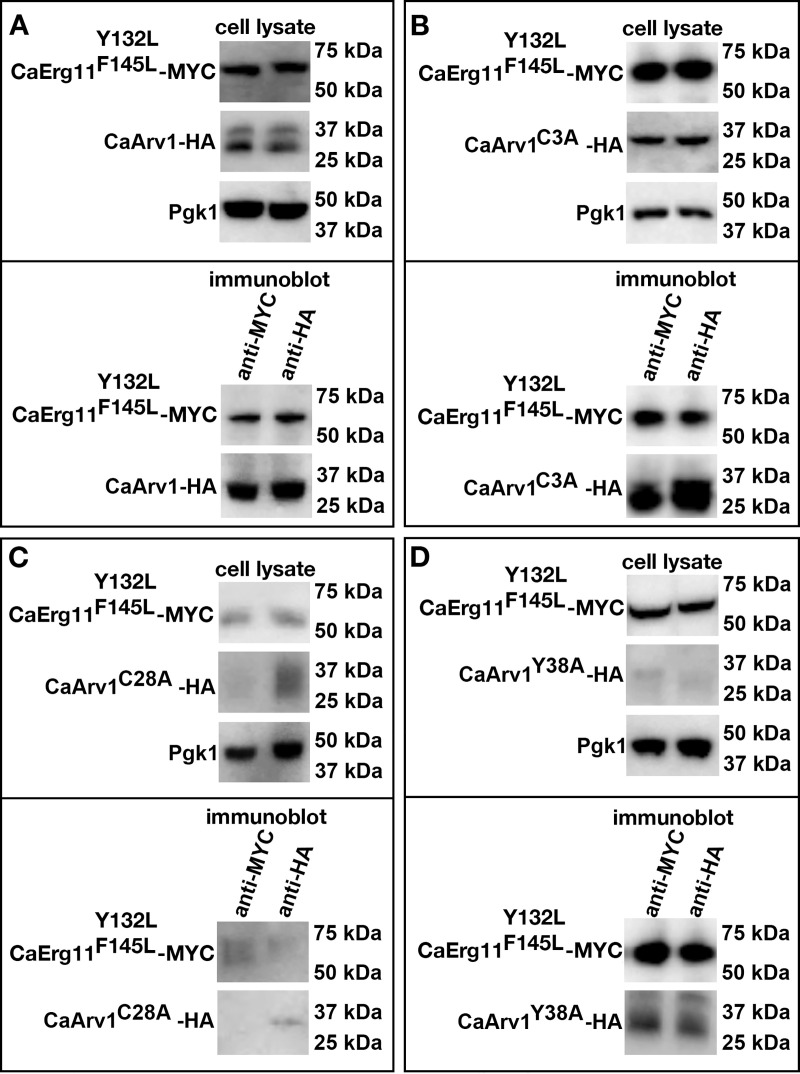
CaArv1^C28A^ is unable to co-immunoprecipitate with CaErg11^Y132L F145L^. *Scarv1* cells co-expressing CaArv1-HA mutants and wild type CaErg11^Y132L F145L^-MYC were grown to exponential phase in the appropriate plasmid selection medium. (A-D), represents the co-immunoprecipitation western results using the indicated anti-MYC antibodies or anti-HA antibodies and indicated Arv1 mutants (immunoblot). The cell lysate panels represent the total Erg11-MYC or Arv1-HA protein levels in total cell lysates. The panels represent data from 3 individual experiments. The differences in detection seen are due to the use of protein lysates from steady state cells. The panels represent data from 3 individual experiments.

We performed co-immunoprecipitation experiments with *Scarv1* cells co-expressing CaArv1-HA, CaArv1^C3A^-HA, CaArv1^C28A^-HA, or CaArv1^Y38A^-HA, and CaErg11^Y132L F145L^-MYC (Fig 9). We found that *Scarv1* cells expressing CaArv1 or CaArv1^C3A^ had similar levels of CaArv1 and CaErg11^Y132L F145L^-MYC (Fig 9A & 9B; cell lysate), whereas, *Scarv1* cells expressing CaArv1^C28A^-HA or CaArv1^Y38A^-HA had reduced levels of CaArv1 and CaErg11^Y132L F145L^-MYC (Fig 9C & 9D, cell lysate). CaArv1-HA, CaArv1^C3A^-HA, and CaArv1^Y38A^ were able to co-immunoprecipitate with CaErg11^Y132L F145L^-MYC (Fig 9A, 9B & 9D, immunoblot). However, CaArv1^C28A^ lacked this ability (Fig 9C, immunoblot).

The possibility does exist that the lack of interaction we see between ScArv1^C27A^-HA and CaArv1^C28A^-HA, and ScErg11-MYC and CaErg11-MYC, respectively, may be due merely to low levels of protein, rather than a loss of a protein-protein interaction. We do not believe this is the case because the expression of CaArv1^Y37A^-HA and CaArv1^Y38A^-HA are similar to ScArv1^C27A^-HA and CaArv1^C28A^-HA, respectively, yet only CaArv1^Y37A^-HA and CaArv1^Y38A^-HA interact with ScErg11-MYC and CaErg11-MYC, respectively. We point out that Erg11 displayed different migration patterns using western analysis. We believe this may due to post-translational modification, such as phosphorylation. We cannot rule this out at this time.

### Scarv1 cells expressing ScArv1^C27A^ accumulate aberrant ER membrane structures

**Fig 10:**
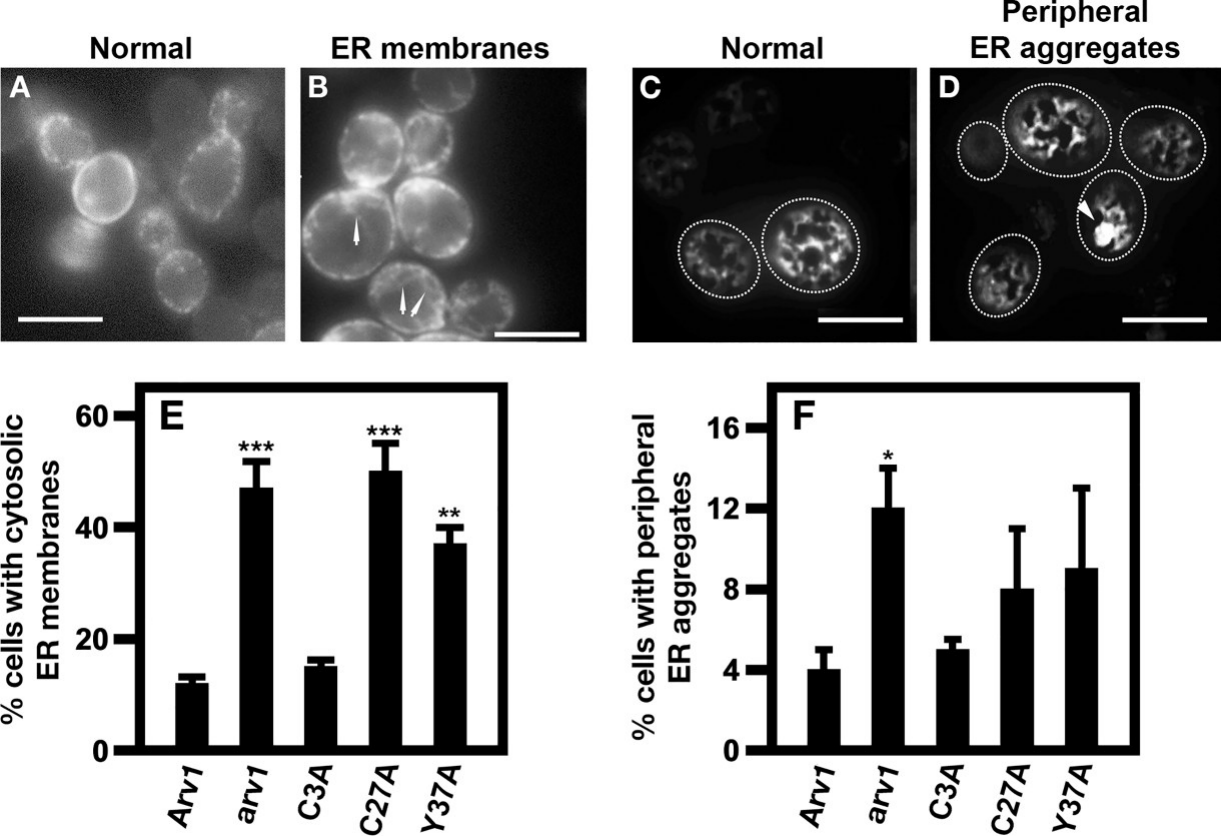
*Scarv1* cells expressing ScArv1^C27A^ accumulates cytosolic ER membrane structures. *S*. *cerevisiae* cells were grown in the appropriate plasmid selection medium. Cells were transformed with *YCplac111-RTN2-GFP* (RTN2 under its own promoter in *CEN/ARS LEU2* vector). For microscopy studies, cells were diluted to approximately OD_600nm_ 0.15. Cells were imaged using a Zeiss AxioImager.Z2 epifluorescence upright microscope with a 100X Plan-Apochromatic 1.4 numerical aperture objective lens. An increase in ER membrane fluorescence at medial (B) or cortical optical sections (D) were manually detected as described in Materials and Methods. (E) Percentage of cells displaying cytosolic ER membranes. (F) Percentage of cells displaying peripheral ER aggregates. 300–500 cells were counted in 3 individual experiments. Arrows in panels B & D point out cytosolic ER membranes and peripheral ER aggregates, respectively. White bar represents 10 um. *p≤ *0*.*01*; **p≤ *0*.*001*; ***p≤ *0*.*0001*.

To further explore what was the reason for the loss of physical interaction between Arv1 and Erg11, we determined if there was a link between defects in ER morphology and loss of protein-protein interaction. We visualized the subcellular localization pattern of the ER resident Rtn2-GFP protein using fluorescence microscopy [36], and based on its subcellular localization pattern, we determined whether cells accumulated cytosolic ER membranes and/or formed ER aggregate structures (Fig 10).

*Scarv1* cells expressing ScArv1 accumulated ~12% cytosolic ER membranes (Fig 10A *vs*. 10B; *normal vs*. *ER membranes*). *Scarv1* cells expressing ScArv1^C3A^ had similar levels, whereas, *Scarv1* cells, and *Scarv1* cells expressing ScArv1^C27A^ or ScArv1^Y37A^, hyper-accumulated these structures (Fig 10A *vs*. 10B; *normal vs*. *ER membranes*). Peripheral ER aggregates only accumulated in *Scarv1* cells to any substantial degree (Fig 10C *vs*. 10D; *normal vs*. *peripheral ER aggregates*. The densitometry units shown in panels 10E and 10F are representative of the percentage of ER membranes and peripheral ER aggregates observed in panels 10B and 10D.

### ScErg11 is mislocalized in *Scarv1* cells expressing ScArv1^C27A^

We next wanted to see if there was a correlation between the defects in ER morphology and the mislocalization of ScErg11. To circumvent the possible reduced protein level of ScErg11-GFP in cells expressing ScArv1^C27A^/CaArv1^C28A^ and ScArv1^Y37A^/CaArv1^Y38A^, which may not allow us to visualize localization, ScErg11 expression was driven by the highly active *GPD* (glyceraldehyde-3-phosphate dehydrogenase) promoter.

**Fig 11:**
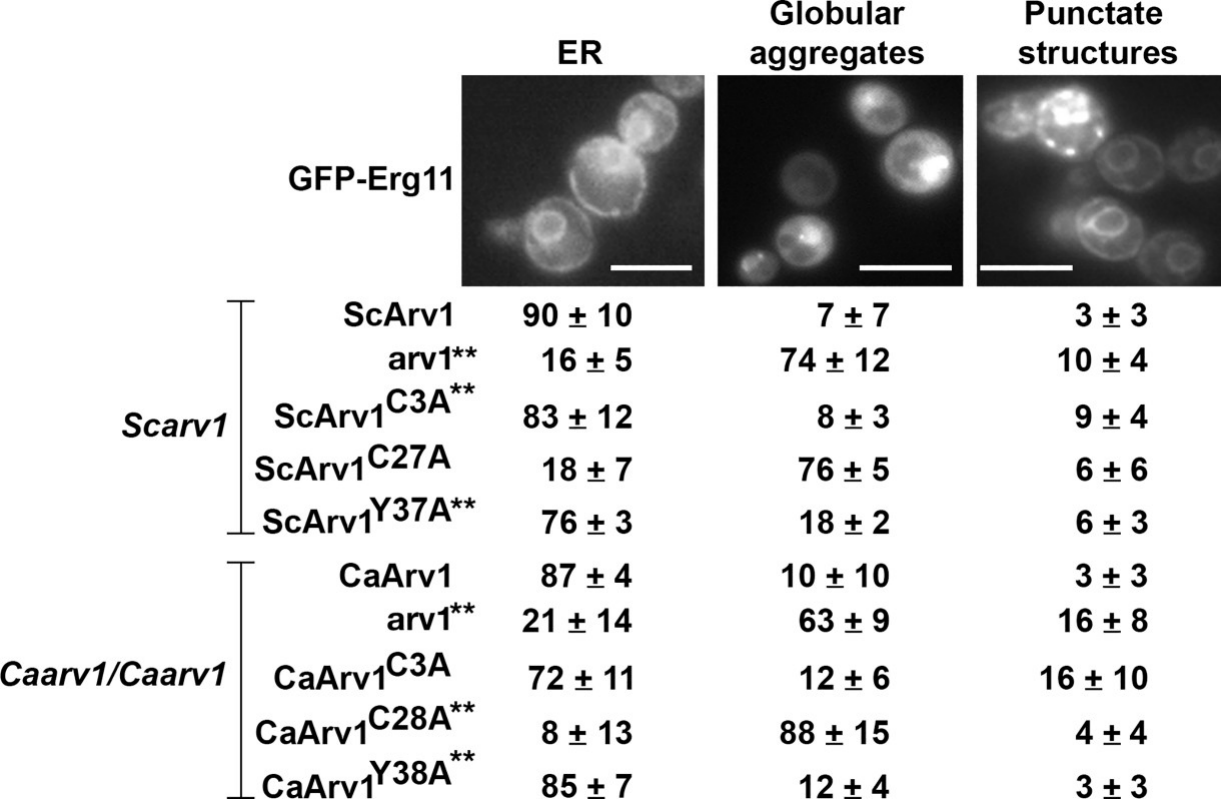
ScArv1 and CaArv1 are required for proper ER localization of GFP-Erg11. GFP-ScErg11 and GFP-CaErg11 proteins, each driven from the *ScGPD* promoter, were expressed in *Scarv1* and *Caarv1/Caarv1* cells, respectively. Cells were grown to exponential phase and GFP-Erg11 was visualized by fluorescence microscopy using a Leica RBME fluorescence microscope and 100X optics. 300–500 cells were counted for each strain using double-blind analysis in 5 individual experiments. The values represent the percentage of cells containing the designated structures. The ER localization of Erg11 has been reported previously [37]. Asterisk indicates cells that display defects in GFP-Erg11 localization.

We found that ScErg11-GFP localized to the ER in *Scarv1* cells expressing ScArv1, ScArv1^C3A^ or ScArv1^Y37A^, whereas it was mislocalized in *Scarv1* cells and *Scarv1* cells expressing ScArv1^C27A^ (Fig 11; asterisk). Similar results were seen in *Caarv1/Caarv1* cells expressing CaErg11-GFP. We saw proper localization of GFP-CaErg11 in *Caarv1/Caarv1* cells expressing CaArv1^C3A^ or CaArv1^Y38A^, while it was mislocalized in cells expressing CaArv1^C28A^ (Fig 11; asterisk).

### *Scarv1* cells expressing ScArv1^C28A^ show constitutive activation of the unfolded protein response

The unfolded protein response (UPR) is constitutively activated is *Scarv1* cells [29]. The reason for this phenotype is unknown, but may be due to accumulation of lipids in the ER. As *Scarv1* cells accumulate lanosterol and unknown sterol intermediates, we asked whether a correlation existed between activation of the UPR and mislocalization of ScErg11. UPR signaling was analyzed by assaying for β-galactosidase activity driven by an integrated unfolded protein response promoter element (UPRE-*LacZ*).

**Fig 12:**
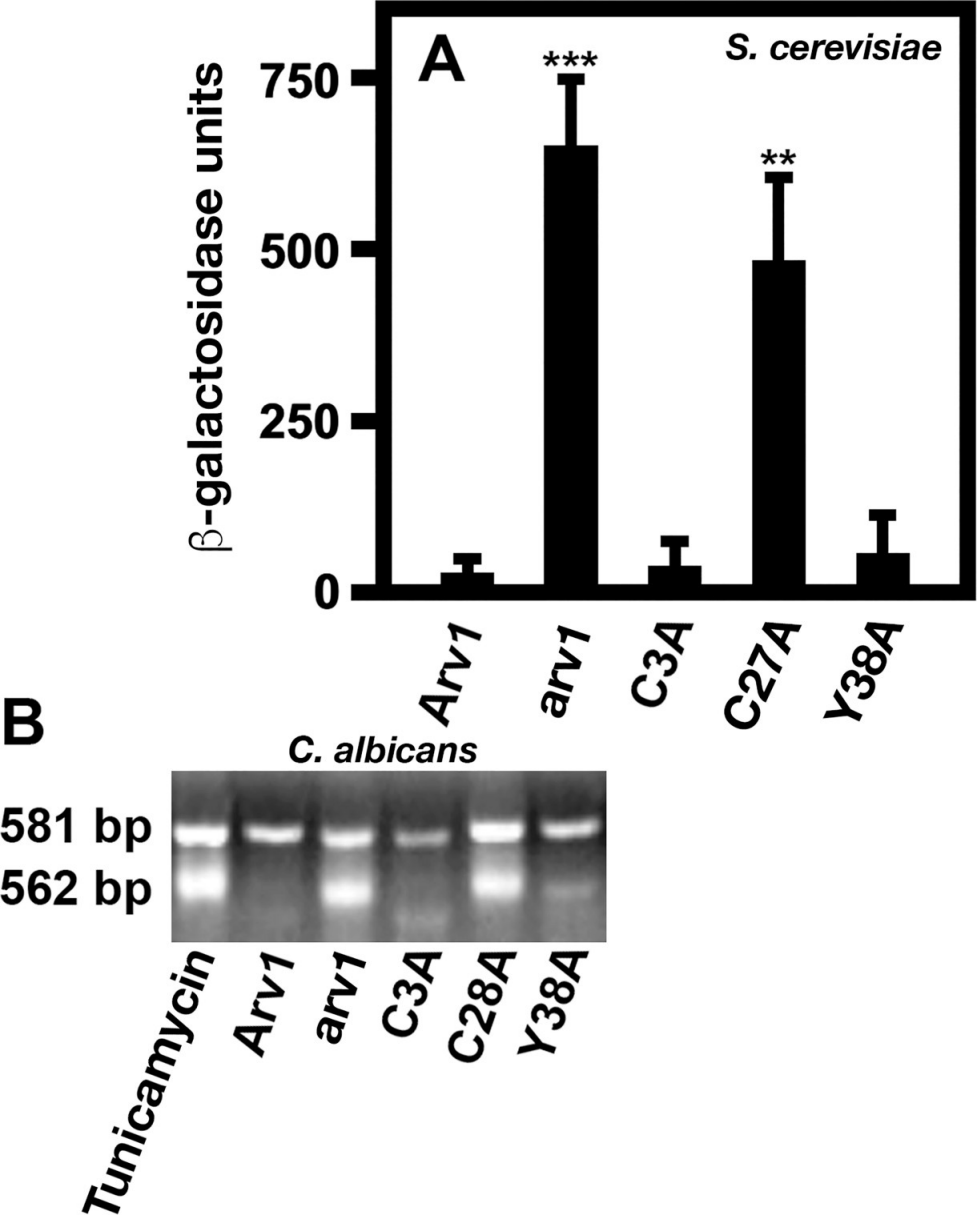
Expression of ScArv1^C27A^ and CaArv1^C28A^ activates the unfolded protein response in *Scarv1* and *Caarv1/Caarv1* cells, respectively. (A) *Scarv1* strains expressing the integrated *UPRE-LacZ* reporter were grown to exponential phase in the appropriate medium. Cells were assayed for the degree of unfolded protein response activation (UPR) by determining β-galactosidase activity. β-galactosidase activity was assayed as described in Materials and Methods. β-galactosidase units were calculated using a previously published equation [38]. (B) The unfolded protein response was determined in *Caarv1/Caarv1* cells by examining the level of *HAC1* mRNA splicing using HAC1-5RT (TGAGGATGAACACCAAGAAGAA) and HAC1-3RT (TCAAAGTCCAACTGAAATGAT) primers and RT-PCR [39]. The levels of unfolded protein activation in various strains were compared to *CaARV1/CaARV1* cells treated with 2μg/ml tunicamycin for 2 hr to activate the UPR (*tunicamycin*). Tunicamycin is an antibiotic that is commonly used to activate ER stress and the UPR. The β-galactosidase results shown in panel A are the average of 5 individual experiments. The agarose gel in panel B is a representative of the results of 5 independent experiments **p≤ *0*.*001*; ***p≤ *0*.*0001*.

We found a direct correlation between the mislocalization of GFP-ScErg11 and an activated UPR (Fig 12A). The UPR was constitutively activated in *Scarv1* and *Scarv1* cells expressing ScArv1^C27A^, whereas it remained at basal levels in *Scarv1* cells expressing ScArv1, ScArv1^C3A^, or ScArv1^Y37A^ (Fig 12A).

We further explored the level of UPR signaling in *Caarv1/Caarv1* cells expressing various mutants. Here, we assayed for the level of *CaHAC1* (**h**omologous to **A**TF/**C**REB1) mRNA splicing [39]. *HAC1* mRNA is spliced from 581 bp to 562 bp when the UPR becomes activated. Tunicamycin treatment of *CaARV1/CaARV1* cells, which activates the UPR, resulted in substantial *CaHAC1* mRNA splicing, indicating that *CaARV1/CaARV1* cells can properly respond upon being stressed (Fig 12B; *tunicamycin*). Under basal conditions in the absence of stress, we did not observe *CaHAC1* mRNA splicing in *Caarv1/Caarv1* cells expressing CaArv1, CaArv1^C3A^, or CaArv1^Y38A^. On the other hand, *Caarv1/Caarv1* and cells expressing CaArv1^C28A^ had a significant increase in the accumulation of the 562 bp spliced *HAC1* mRNA, indicating that the UPR was constitutively activated (Fig 12B). The degree of splicing in these cells was similar to that for *CaARV1/CaARV1* cells treated with tunicamycin (Fig 12B; *tunicamycin*).

### *Carv1/Caarv1* cells expressing CaArv1^C28A^ are hypersusceptible to azole antifungal drugs

To determine if the loss of a CaErg11-CaArv1 interaction in *C*. *albicans* cells affects azole susceptibility, we grew *Caarv1/Caarv1* cells expressing CaArv1^C3A^, CaArv1^C28A^, or CaArv1^Y38A^, in the presence of itraconazole or fluconazole, and we determined MIC concentrations. We found that *Caarv1*/*Caarv1* cells were hypersusceptible to both antifungal drugs (Table 2). *Caarv1/Caarv1* cells expressing CaArv1^C28A^ were also hypersusceptible to either antifungal (Table 2), while cells expressing CaArv1^C3A^ or CaArv1^Y38A^ retained normal susceptibility. Interestingly, expressing Erg11^Y132L Y145L^ in *Caarv1*/*Caarv1* cells expressing CaArv1^C28A^ did not restore normal susceptibility to itraconazole or fluconazole. Nor did it result in resistance when co-expressed in *Caarv1/Caarv1* cells expressing CaArv1^C3A^ or CaArv1^Y38A^.

**Table 2:** Antifungal susceptibilities of *Caarv1/Caarv1* mutant strains.

Strain	Itraconazole	Fluconazole
*BWP17 (WT)*	0.44 ± 0.13	12.5 ± 2.6
*Caarv1/Caarv1*	0.08 ± 0.01**	1.4 ± 0.3**
*Caarv1/Caarv1 + CaARV1*	0.57 ± 0.17	16 ± 3.1
*Caarv1/Caarv1 + CaARV1*^*C3A*^	0.49 ± 0.03	11.3 ± 0.25
*CaArv1/Caarv1 + CaARV1*^*C28A*^	0.02 ± 0.07***	0.94 ± 0.05***
*Caarv1/Caarv1 + CaARV1*^*Y38A*^	0.53 ± 0.07	14.8 ± 1.6
*Caarv1/Caarv1 + CaARV1*^*C3A Y132L Y145L*^	0.64 ± 0.26	10.90 ± 2.4
*CaArv1/Caarv1 + CaARV1*^*C28A Y132L Y145L*^	0.04 ± 0.03***	3.7 ± .086***
*Caarv1/Caarv 1 + CaARV1*^*Y38A Y132L Y145L*^	0.71 ± 0.11	15.8 ± 1.03

^a^values are μg/ml.

***p<0.0001.

### *Caarv1/CaArv1* cells co-expressing CaArv1^C28A^ and CaErg11^Y132L F145L^ lack virulence

Finally, we determined whether the loss of interaction between CaArv1^C28A^ and CaErg11 correlated with a lack of virulence in a disseminated candidiasis murine model. We constructed *Caarv1/Caarv1* strains expressing CaArv1, CaArv1^C3A^, CaArv1^C28A^, CaArv1^Y38A^, or strains co-expressing CaErg11^Y132L F145L^ with CaArv1^C3A^, CaErg11^Y132L F145L^, or CaArv1^C28A^. We injected BALB/C mice with each strain and monitored survival for 30 days.

**Fig 13:**
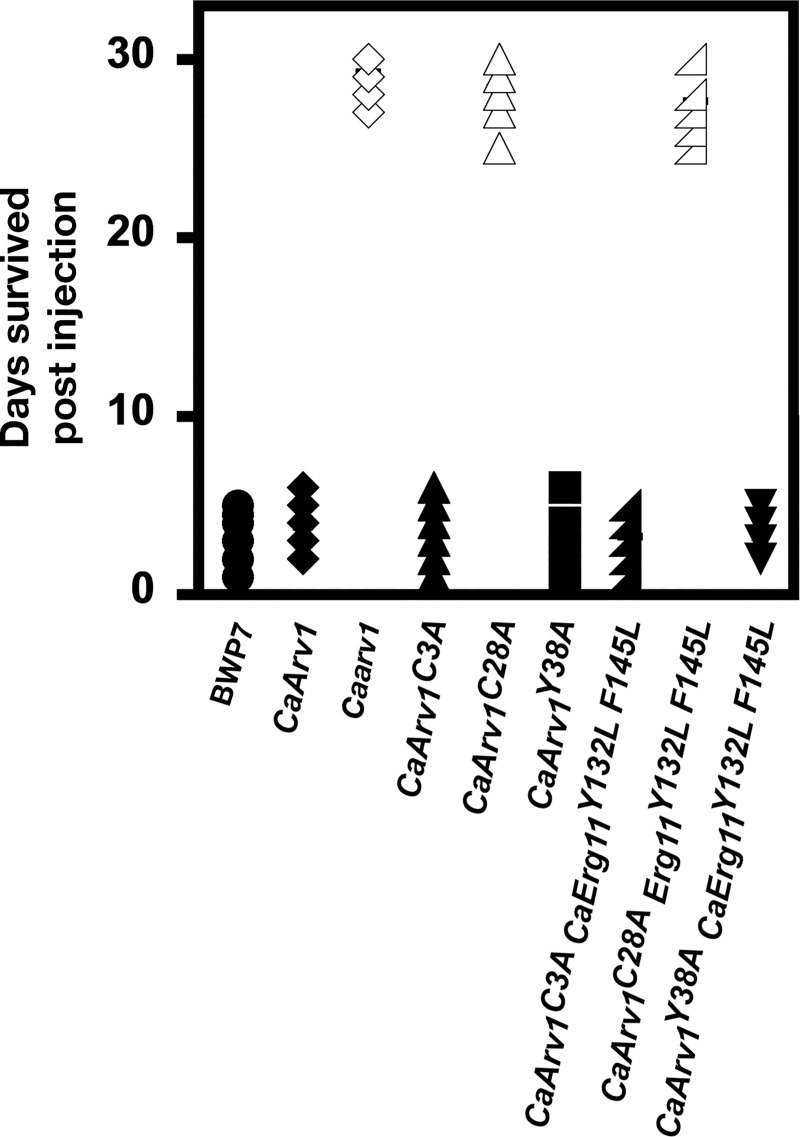
*Caarv1/Caarv1* cells expressing CaArv1^C28A^ lack virulence. BALB/C mice were tail vein injected with *Caarv1/Caarv1* cells expressing CaArv1^C3A^, CaArv1^C28A^, CaArv1^Y38A^, or *Caarv1/Caarv1* cells co-expressing CaArv1^C3A^ and Erg11^Y132L F145L^, CaArv1^C28A^ and Erg11^Y132L F145L^, or CaArv1^Y38A^ and Erg11^Y132L F145L^. Viability was examined daily for 30 days. Mice injected with *Caarv1/Caarv1* expressing CaArv1^C28A^ remained viable during the entire time of the study, whereas mice injected with *Caarv1/Caarv1* expressing CaArv1^Y38A^ died within a few days. Mice injected with CaArv1^C28A^ and Erg11^Y132L F145L^ also died within a few days.

We found that mice injected with *CaARV1/CaARV1*, *Caarv1/CaARV1*, *Caarv1/CaARV1*^*C3A*^, and *Caarv1/ARV1*^*Y38A*^ cells all died within 6 days (Fig 13). In contrast, mice injected with *Caarv1/Caarv1* or *Caarv1/CaARV1*^*C28A*^ cells remained viable for the entire time of the study. Interestingly, we found that mice injected with *Caarv1/Caarv1* cells expressing CaErg11^Y132L F145L^ also remained viable for the entire study, suggesting that CaErg11^Y132L F145L^ expression was unable to eliminate the deleterious effect of losing CaArv1. Finally, we observed that *Caarv1/Caarv1* cells co-expressing CaArv1^C28A^ and CaErg11^Y132L F145L^ also lacked virulence, while *Caarv1/Caarv1* cells expressing CaArv1^Y38A^ and CaErg11^Y132L F145L^ retained virulence.

## Discussion

Fungal infections caused by azole resistant *Candida spp*. are a major health concern for the immune compromised. Azole-resistant *Candida spp*. are emerging in the clinic. Here, we uncovered that Cys27 within the ScAhd is required for normal azole susceptibility, proper sterol synthesis, and ScErg11 stability, all of which require that ScArv1 and ScErg11 interact and form a complex. *Scarv1* cells expressing ScArv1^C27A^ were azole hypersusceptible, harbored reduced levels of ScErg11, and accumulated the Erg11 substrate, lanosterol. In addition, ScArv1^C27A^ was unable to co-immunoprecipitate with ScErg11. *Scarv1* cells expressing CaArv1^C28A^ displayed the same phenotypes. Importantly, *Caarv1/Caarv1* cells expressing CaArv1^C28A^ lacked virulence, and expressing CaErg11^Y132L Y145L^ in these cells was unable to restore virulence in a mouse model of candidiasis. These results strongly suggest that one function of Arv1 is to interact with and stabilize Erg11. This interaction may be required for the virulence of *C*. *albicans* cells.

Initially, we focused our efforts on defining ScArv1 domains required for maintaining normal azole susceptibility and sterol synthesis. We found that neither the ScAhd nor ScΔAhd could replace full-length ScArv1. As there is little to no homology between the ScΔAhd of other *Candida spp*., we believe that ScΔAhd is only needed to tether the ScAhd to the ER membrane.

We chose to focus on defining important conserved amino acids within the ScΔAhd that were required for Arv1 function. While alanine substitution did cause reductions in protein levels of certain ScArv1 mutants, we did not observe a direct correlation between loss of detectable protein level and hypersusceptibility to azole treatment (Fig 1; Table 1). In particular, our results obtained using *Scarv1* cells expressing ScArv1^C27A^ and ScArv1^Y37A^. Both proteins were expressed at low levels, but *Scarv1* cells expressing ScArv1^C27A^ remained hypersusceptible to azoles, while *Scarv1* cells expressing ScArv1^Y37A^ showed a normal response.

Interestingly, *Scarv1* cells expressing ScArv1^C3A^ or ScArv1^Y37A^ had defects in sterol composition and localization. However, *Scarv1* cells expressing ScArv1^Y37A^ had a normal UPR, whereas *Scarv1* cells expressing ScArv1^C27A^ showed a constitutive activation. This suggests that ScArv1^Y37A^ expressing cells are able to retain some level of ER membrane integrity. This may help to explain why ScArv1^Y37A^ was able to interact with ScErg11, and that ScErg11 properly localized to the ER in *Scarv1* cells expressing this protein. We found similar results for *Caarv1*/*Caarv1* cells expressing CaArv1^C28A^ and CaArv1^Y38A^. Table 3 summarizes the phenotypes of *Scarv1* cells expressing ScArv1^C27A^ or ScArv1^Y37A^.

**Table 3:** Phenotypes of Arv1 proteins.

Phenotype	ScArv1	Scarv1	ScArv1^C27A^	ScArv1^Y37A^
Normal steady-state Arv1 protein level	+++	-	-	-
Normal sterol composition	+++	-	-	-
Normal sterol distribution	+++	-	-	-
Normal steady-state Erg11 level	+++	-	-	-
Erg11 stabilized by MG132	+++	++	++	++
Positive interaction in Y2H	+++	-	-	+++
Interacts with Erg11	+++	-	-	+++
Interacts with Erg11^Y132L L145R^	+++	-	-	+++
Cytoplasmic ER membranes	-	+++	+++	++
ER aggregates	-	+++	-	-
Proper localization of Erg11	+++	-	-	+++
Activated UPR	-	+++	+++	-
virulence	+++	-	-	+++

Cys27 is found within the ScAhd zinc-binding domain, as is Cys28 in *C*. *albicans*. Both are part of a cysteine cluster (two CXXC motifs separated by ~20 amino acids) that is conserved across *Candida spp*. Many transcription factors have zinc-binding motifs that are involved in DNA binding [40]. Moreover, this same motif has been shown to be required for protein-protein interactions [41, 42]. There is no evidence to date that suggests that Arv1 has any role in directly regulating gene expression. It may however be needed for maintaining the stability of certain Erg proteins, possibly through direct interaction. We found that ScArv1 was required for ScErg1 and ScErg25 protein stability, but not ScErg6 or ScErg24. Based on these data, our hypothesis is that the Ahd of ScArv1 may act as a scaffolding domain that is involved in maintaining the stability of a subset of Erg proteins. Thus, it may function, along with the ScErg28 scaffold, to stabilize the ergosome complex [43]. This function may be conserved in *C*. *albicans*.

The addition of the proteosome inhibitor, MG132, stabilized ScErg11 in *Scarv1* cells expressing ScArv1^C27A^. ScErg11 was also stabilized in *Scarv1* cells lacking the proteosomal pre1/2 chymotrypsin-like enzymes, as well as cells lacking Doa4, which is involved in ubiquitin recycling. Based on these data, our hypothesis is that the loss of Arv1 causes accelerated degradation of ScErg11 through an ubiquitin-dependent mechanism, which ultimately targets it for degradation by the proteosome. There is evidence other ScErg proteins are ubiquitinated. Peng et al., [8] used a proteomic approach to identify ubiqutinated sites within the yeast proteome. They found that ScErg11 was enriched in the ubiquitinated fraction and contained a putative ubiquitination site. Other ScErg proteins that were enriched in this same fraction included ScErg1/9/26/27. They also definitively confirmed the presence of multiple ubiquitination sites within ScErg3 and ScErg5. These results suggest that ubiquitin-dependent degradation of ScErg proteins may be a common mechanism used to maintain proper sterol biosynthesis.

**Fig 14:**
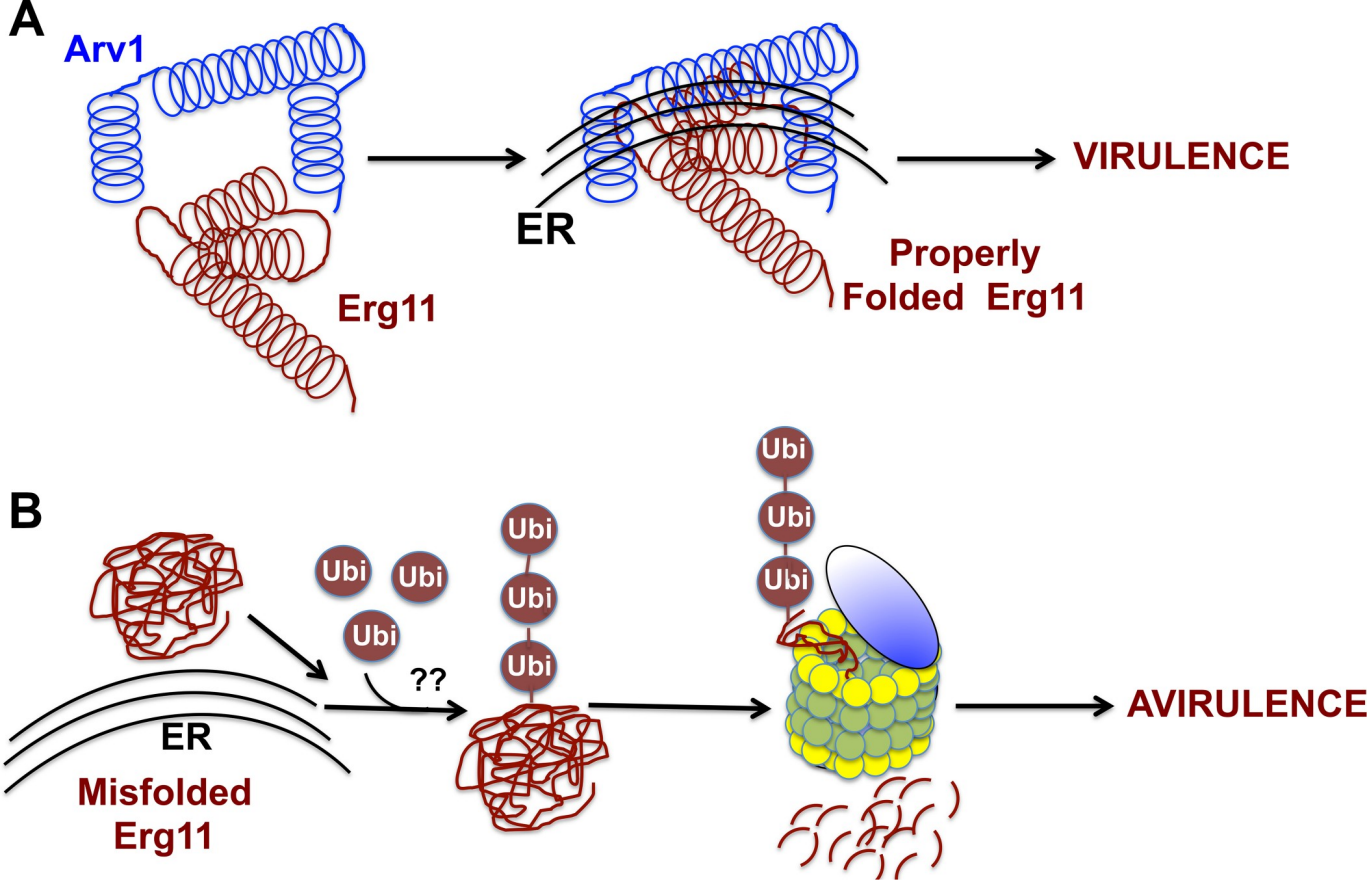
Model depicting the role of Arv1 in maintaining Erg11 stability and virulence. (A) A direct physical interaction between Arv1 and Erg11 results in stability of Erg11. This interaction may be required for maintaining Erg11 tertiary structure and/or tether it to the ER membrane. A stable Erg11-Arv1 complex confers virulence. (B) Erg11 becomes misfolded in the absence of Arv1, and may be targeted to the proteosome via ubiquitination. Cells lacking a stable Erg11-Arv1 complex are azole hypersusceptible and lack virulence.

Based on our data, we have come up with a model for regulation of Erg11 stability and function (Fig 14). It is assumed that all *arv1* mutants contain some level of Erg11, as *erg11* null cells are inviable. We believe that Arv1, through its interaction with Erg11, aids in retaining Erg11 in the ER, which is necessary for proper folding and function (Fig 14A). The absence of Arv1 results in the mislocalization of ScErg11, which leads to its accelerated degradation by the proteosome (Fig 14B). Degradation may or may not be driven by Erg11 polyubiquitination. Erg11 degradation would presumably lead to accumulation of sterol in the ER, in particular, lanosterol. Sterol accumulation may initiate or exasperate the degree of UPR activation. It is presumed that multiple ER membrane-associated proteins would also be mislocalized by loss of Arv1, thus it may not solely be the misfolding of ScErg11 that causes UPR activation. We show here that ScErg1 and ScErg25 levels were reduced in *Scarv1* cells, but were stabilized by the addition of MG132. Overall, we suggest that the lack of virulence phenotype of *Caarv1/Caarv1* cells is partly due to severely reduced CaErg11 levels brought on by loss of Arv1, explaining why these cells are hypersusceptible to azoles.

We are aware that reduced protein expression of certain Arv1 mutants may come into affect when indicating whether or not there is an interaction between a specific Arv1 mutant and Erg11. The interaction may be weak and below the detection level of western analysis. There is also the probability that these mutants may be partially misfolded and this reduces their affinity to Erg11. However, our data does show that certain Arv1 mutants that are expressed at lower levels show different affinity for binding to Erg11.

In addition to lanosterol and unknown sterol intermediates, *Scarv1* cells also accumulate several ceramide species [26]. Whether ceramide accumulation plays a role in ScErg11 protein instability or localization, and/or the appearance of any or all *Scarv1* phenotypes, especially the perturbed membrane morphology observed, is not known. Ceramide synthesis occurs in the ER, the organelle whose morphology is distorted in *Scarv1* cells [44]. Ceramide hyper-accumulation and its mislocalization is seen in Niemann-Pick type C [45], Gaucher disease [46], and cystic fibrosis [47] patients, and is a contributor to the progression of these diseases. It remains to be examined if and how ceramide accumulation affects sterol metabolism.

The function(s) of Arv1 remains unresolved. There is accumulating evidence supporting the idea that Arv1 may be a lipid-binding protein in mammalian and *S*. *cerevisiae* cells, although this hypothesis has been challenged with regards to the yeast protein [28]. Concerning CaArv1 function, our data strongly suggests that CaArv1 plays an important role in virulence through its interaction with, and stabilization of, CaErg11. Thus, we believe that fungal Arv1 may represent a new target for inhibiting the invasion of multiple pathogenic fungi, especially those found to be azole resistant.

## Materials and methods

### Strains, media, and miscellaneous microbial techniques

Yeast strains (W303α, *S*. *cerevisiae*; BWP7, *C*. *albicans*) (S1 & S2 Tables) were grown in either YEPD (1% yeast extract, 2% bacto-peptone, 2% glucose), or synthetic minimal medium containing 0.67% Yeast Nitrogen Base (Difco, Sparks, MD) supplemented with the appropriate amino acids, adenine, and uracil. All antifungals were added to liquid cultures. Yeast transformation was performed using the procedure described by Ito *et al*., [48]. For routine propagation of plasmids, *E*. *coli* XL1-Blue cells were used and grown in LB medium supplemented with ampicillin (150 μg/ml).

### Plasmid construction and site-directed mutagenesis

The *S*. *cerevisiae* strains used in our studies were derived from W303-1A (*MAT***a** *ura3 leu2 his3 trp1 ade2*); the designated wild-type strain. *pRS416-HA-URA3-CEN* was used as a template to generate plasmids expressing various ScArv1-HA and CaArv1-HA fusion proteins in *S*. *cerevisiae*. Protein expression in *S*. *cerevisiae* was driven from the endogenous *ScARV1* promoter (-1,000 to 0). *pRS416-HA-URA3-CEN* was selected for by plating on Ura^-^ synthetic medium.

All ScArv1 point mutants were generated using the Stratagene QuikChange® Site-Directed Mutagenesis Kit (Santa Clara, CA). Modifications to the PCR protocol included extending the elongation cycle from 1m/kb to 2m/kb and increasing the number of cycles to 30. *pRS416-ScARV1-HA-URA3-CEN* was used as a template for constructing point mutants.

CaArv1 point mutations were generated using the QuickChange Site Directed Mutagenesis Kit (Stratagene) and *pDDB78-HIS1-CaARV1* (*pHIS1*) as a template. Protein expression was driven from the endogenous *CaARV1* promoter.

The low copy centromeric plasmid *pRS415-MYC-LEU2-CEN* was used to express ScErg11-MYC, CaErg11-MYC, and CaErg11^Y132L F145L^-MYC. Gene expression was driven by *ScERG11* endogenous *S*. *cerevisiae* promoter sequences (-1,000 to 0). *pRS415-MYC-LEU2-CEN* was selected for by growing cells in Leu^-^ medium. When *pRS416-ScARV1-HA-URA3-CEN* and *pRS415-MYC-LEU2-CEN* were co-expressed, cells were grown in Ura^-^ Leu^-^ double drop out medium.

The *pRS415-GAL1-GPD-MYC-LEU2* plasmid was used for high-level inducible expression of ScErg-MYC and was selected for by growing strains in Leu^-^ medium. The pCB74-TRP1-2μm plasmid was used to overexpress GFP-Erg11, and was selected for by growing strains in Trp^-^ medium [26].

The *C*. *albicans* strain BWP17 (*ura3/ura3 arg4/arg4 his1/his1*) was used to generate all strains; designated as the wild type strain. All *C*. *albicans* strains were grown in YEPD medium. The *Caarv1/Caarv1* strain was generated as previously described using the *pGEM-URA3* and *pRS-ARG4ΔSpeI* plasmids [25, 49, 50]. To integrate wild type and *CaARV1* mutant alleles into the *Caarv1/Caarv1* strain, they were first PCR amplified and PCR fragments were cloned into the disruption plasmid, pDDB78-*HIS1* [51]. pDDB78-*HIS1* plasmids containing CaArv1 mutant alleles were integrated into *Caarv1/Caarv1* cells at the *HIS1* locus. Integration was verified by PCR amplification through the *HIS1* locus. Transformants were selected on medium lacking uracil, arginine, and histidine. Integration was subsequently verified by PCR. *pRS-ARG4ΔSpeI* was used for integration of the *CaErg11*^*Y132l F145L*^ allele at a single *CaErg11* allele. The Clp10 vector was use for integration at the *URA3* locus [52]. Thus, all strains were integrated with all selectable markers (*URA3*, *ARG4*, and *HIS1*) to eliminate auxotrophic effects.

GFP-CaErg11 was integrated into various homozygous and heterozygous *Caarv1/Caarv1* strains by allele integration at the *CaARV1* locus using *pGEM-URA3* and/or *pRS-ARG4ΔSpeI* plasmids. Briefly, *pCLP10-URA3* and *pRS-ARG4ΔSpeI* plasmids were constructed to contain GFP-*ERG11* sequences from pCB74, which included the *GPD* promoter and GFP sequences, flanked by 70 bp of *CaARV1* sequence. Integration results in the simultaneous disruption of the *CaARV1* locus with its replacement with the *GFP-CaERG11* allele.

*S*. *cerevisiae* strains used for assaying UPR activation were generated by integrating the YIp integrating vector, *pRS406-UPRE-LacZ* [53]. *pRS406-UPRE-LacZ* contains an *UPRE* promoter sequence (CAGCGTG) fused to *LacZ* [38, 54]. *pRS406-UPRE-LacZ* integration was accomplished by vector digestion with *Stu1* and transformation into cells. Positive clones were selected on Ura^-^ synthetic plates.

### Protein extraction and immunoblotting

Total protein was isolated using the NaOH/TCA cell lysis/protein extraction method. Briefly, cells were snap frozen in liquid N_2_, and lysed in 1.85M NaOH + 7.4% β-mercaptoethanol. Protein was precipitated from cell lysis solution in 25% trichloroacetic acid (TCA), washed with acetone, and resuspended in 5% SDS in 0.1M Tris Base. Epitope-tagged Arv1 constructs were detected using an anti-HA monoclonal antibody (clone 16B12) from Covance (Princeton, NJ). To detect 3HA tagged Arv1, Immobilon Western Chemiluminescent HRP substrate (Millipore, Billerica, MA) was used as substrate for HRP-conjugated secondary mouse antibodies (GE Healthcare, Waukesha, WI). Ponceau S staining and the Bradford method were used in order to load equal amounts of protein in each well. ScPgk1 was used as a loading control.

To visualize Erg11-MYC, cell pellets were resuspended in lysis buffer (0.2M Tris Base, 0.39M (NH_4_)_2_SO_4_, 10mM MgSO_4_, 20% v/v Glycerol, 1mM EDTA, 1 mM AEBSP, Roche protease inhibitor cocktail (Roche Applied Science, Indianapolis, IN), pH 7.9) and ice-cold glass beads. Cells were then vortexed at 4°C for 1 min and put on ice for 1 min; this was repeated for a total of 10 times. The lysate was centrifuged at 3,000 X g for 5min. Supernatants were collected and assayed for protein concentration using the Bradford reagent (Bio-Rad, Hercules, CA). Erg11-MYC was detected using an anti-MYC monoclonal antibody (clone 9E10; 1:1000 dilution) from Covance (Princeton, NJ). Pgk1 was used as a loading control (anti-Pgk1, Invitrogen, Carlsbad, CA; 1:2,500 dilution). To detect Erg11-MYC and Pgk1, Pierce ECL Western blotting substrate (Thermo Scientific, Waltham, MA) was used as a substrate for HRP-conjugated secondary mouse antibodies (GE Healthcare).

### Minimal inhibitory concentration (MIC) assay

All strains were grown to exponential phase (0.5_OD630_) in the appropriate synthetic plasmid selection medium. All proteins were expressed using low copy vectors and expression was driven from the endogenous *ARV1* promoter. 100 μl of 1x10^4^ cells/ml of each strain were plated into 96-well plates. 2-fold dilutions of antifungal drug were added and MICs were determined after 24 hr growth at 30°C. MICs were characterized as the lowest concentration causing ~50% inhibition of growth at 24 hr, as measured at an OD_630_. To further validate the MIC values, plate dilutions were also performed and viable cells were counted. We found either method gave similar MIC results. All antifungal drugs were purchased from Sigma-Aldrich (St. Louis, MO) and solubilized in DMSO.

### Sterol composition analysis

Sterol analysis was performed as previously described [21].

### Sterol localization analysis

The localization of unesterified sterol was visualized using filipin staining. Cells were grown to exponential phase in synthetic medium. Cells were fixed with 3.7% formaldehyde for 10 min at 23°C under constant mixing. Fixed cells were washed twice with distilled water. Cells were then incubated with 100μg/ml filipin (Sigma Chemicals) in the dark for 15 min at 23°C. Filipin fluorescence was visualized using a Leica DMR fluorescence microscope and UV optics.

### mRNA stability assay

mRNA decay was determined using 3 μg/ml thiolutin as described [55].

### ScErg11-MYC protein stability assay

Cells expressing ScErg11-MYC under the *GAL1* promoter were grown to exponential phase in 2% raffinose. 2% galactose was added to cultures for 2 hr to induce ScErg11-MYC expression. 2% glucose was subsequently added and cells were collected at the indicated times.

### MG132 assays

Cells were grown to exponential phase. 35 μg/ml of cyclohexamide was added to all cultures. Cells were then incubated with and without MG132 (75 mM) for 45 min. Cell pellets were collected, and ScErg11 protein levels were determined by western analysis and immunoblotting as described below.

### Split-ubiquitin two-hybrid assay

The L40 (*MATa trp1 leu2 his3 LYS2*::*lexA-HIS3 URA3*::*lexA-lacZ*) yeast strain was used for two-hybrid studies. L40 contains *HIS3* and *LacZ* reporter genes under the control of the lexA promoter. A protein A-lexA-VP16 (PLV) fragment is released upon membrane protein–protein interaction, freeing it up to activate these promoters. The Cub vector (pCYC-BAIT-Cub-PLV) and Nub vector (pADH-PREY-2HA-NubG) were used for expressing various Arv1 and Erg11 proteins, and determining the level of protein-protein interaction. They were co-transformed in L40 and double transformants were selected for and grown on Leu-Trp- medium to maintain plasmid selection. The degree of protein-protein interaction was assayed by determining the robustness of growth on Leu-Trp-His- medium and level of β-galactosidase activity over the negative control.

### Co-immunoprecipitation assay

Cells were grown to exponential phase in the appropriate medium to maintain plasmid selection. Cells were pelleted and resuspended in lysis buffer (200 mM Tris-HCl, pH 8.0, 400 mM (NH_4_)_2_SO_4_, 10 mM MgSO_4_, 20% v/v glycerol, 1 mM EDTA) plus protease inhibitors (Sigma). 1 mg of total protein was resuspended in 500 μl in buffer A. Cells were then incubated with the appropriate antibody ((anti-HA monoclonal antibody (clone 16B12), 10 μl; anti-MYC monoclonal antibody (clone 9E10); 10 μl)) for 4 hr at 4°C. 20 μl of Protein A agarose beads was then added to the antibody/cell lysate slurry and incubated for 4 hr at 4° C. Complexes were pelleted and washed several times with Co-IP buffer (10 mM HEPES, pH7.5, 200 mM NaCl, 5 mM EDTA, 0.1% Nonidet P-40), and subsequently resuspended in treatment buffer, and proteins were resolved using western analysis as described below.

### ER retention studies

Cells were grown overnight in synthetic medium lacking uracil and transformed with YCplac111-RTN2-GFP (*RTN2* driven by its endogenous promoter in a *CEN/ARS LEU2* vector). Transformants were selected on plates lacking uracil and leucine.

### Fluorescence microscopy

For microscopy studies, cells were grown overnight in synthetic medium lacking uracil and leucine and diluted to an OD_600_ of 0.15. Cells were grown for at least 7 h before microscopy. Cells were imaged live at room temperature using a Zeiss AxioImager.Z2 epifluorescence upright microscope with a 100X Plan-Apochromatic 1.4 numerical aperture (NA) objective lens (Carl Zeiss Ltd, Jena, Germany). Images were recorded using a large chip sCMOS mono camera for sensitive fluorescence imaging (ORCA Flash 4.0v2, Hamamatsu, Hamamatsu, Japan), saved by Zeiss ZEN2.3 software (Blue edition, Carl Zeiss Ltd, Jena, Germany) and exported to Adobe Photoshop (Adobe, San Jose, CA). Through-focus image series were deconvolved with Volocity 6.3 (PerkinElmer, Waltham, MA) using calculated point-spread functions. Stacks were then visualized using ImageJ 1.47v (NIH, Bethesda, MD) to manually detect membrane aggregates at the periphery of the cell or increased cytosolic ER membranes in the cytosol at medial optical sections, by comparing the reporter fluorescence in the wild type cells with mutant cells. Except for the wild type strain, which was used as a reference, quantification of ER membranes was performed blindly.

### UPRE-LacZ promoter driven β-galactosidase assay

β-galactosidase activity assays were performed as described using the substrate chlorophenol red-β-D-galactopyranoside [38].

### *HAC1* mRNA splicing assay

*Caarv1/Caarv1* Cells were grown to exponential phase. To measure the level of *HAC1* mRNA splicing, total RNA was obtained using a RNA extraction kit (Promega, USA) and was used to generate a cDNA pool by reverse-transcription using an Oligo(dT)-primed RT reagent Kit (Promega, USA). HAC1-5RT and HAC1-3RT primers were used to amplify these cDNA by PCR (HAC1-5RT (TGAGGATGAACACCAAGAAGAA), and HAC1-3RT (TCAAAGTCCAACTGAAATGAT) [39]. PCR products were separated by 3% agarose gel electrophoresis. *CaARV1/CaARV1* cells were treated with 2μ/ml tunicamycin for 2 hr as a control to activate the unfolded protein response.

### Disseminated candidiasis studies

The disseminated candidiasis mouse model was used for all virulence studies. Male BALB/c mice (Jackson Labs) ages 6–8 weeks, weighing approximately 18-22g, housed in groups of as many as four animals, were supplied food and water *ad libitum*. Cells were grown in YEPD and injected via the tail vein with 200 μl of 1X10^6^ cells/ml in 1X PBS. Infected animals were monitored daily for up to 30 days post-infection and were considered moribund when they had a ruffled coat, were hypothermic, and could no longer reach food or water. Animals were euthanized by CO_2_ asphyxiation and survival times were recorded. All experiments were carried out according to the NIH guidelines for the ethical treatment of animals.

### Statistical analysis

Student’s t-test and one-way anova with Bonferroni post hoc were used to determine significance of the data.

### Ethics statement

The Temple University IACUC approved the animal use protocol (#3284). Standards for the ethical treatment of mice follow the *Guide for the Care and Use of Laboratory Animals* (*Guide*), Public Health Service (PHS) Policy (Assurance with the Office of Laboratory Animal Welfare (OLAW)), which follows the standards set forth by the *Guide* and PHS Policy for all animals, and the Animal Welfare Act (AWA) regulations for covered species. In addition, treatment follows the regulations and guidelines set forth by the U.S. Department of Agriculture (USDA). All animals regardless of USDA covered species or PHS funded studies are treated the same.

## Supporting information

S1 Table *S*. *cerevisiae* strains. (DOCX)

S2 Table *C*. *albicans* strains. (DOCX)

S1 Raw image (TIF)

S2 Raw image (TIF)

S3 Raw image (TIF)

S4 Raw image (TIF)

S5 Raw image (TIF)

S6 Raw image (TIF)

S7 Raw image (TIF)

S8 Raw image (TIF)

S9 Raw image (TIF)
