# Supplementary material for: An Erg11 lanosterol 14-α-demethylase-Arv1 complex is required for Candida albicans virulence
Source: PLoS One. 2020 Jul 17;15(7):e0235746. doi: 10.1371/journal.pone.0235746 (PMC7367482; doi:10.1371/journal.pone.0235746)
Supplement: S1 Table — (DOCX) [file pone.0235746.s001.docx]

Table S1 *S. cerevisiae* strains

| *S. cerevisae* | | |
| --- | --- | --- |
| Strains | Genotype | Plasmid |
| W303-1A (WT)  *ScARV1* | ****a*** ura leu2 trp1 ade2* |  |
| *Scarv1* | ****a*** ura leu2 trp1 ade2 ARV1::Kan^r^* |  |
| *Scarv1 + Arv1* | ****a*** ura leu2 trp1 ade2 ARV1::Kan^r^* | *pRS416-ARV1-HA-URA3-CEN* |
| *Scarv1 + Arv1^C3A^* | ****a*** ura leu2 trp1 ade2 ARV1::Kan^r^* | *pRS416-ARV1 ^C3A^ -HA-URA3-CEN* |
| *Scarv1 + Arv1^C6A^* | ****a*** ura leu2 trp1 ade2 ARV1::Kan^r^* | *pRS416-ARV1 ^C6A^ -HA-URA3-CEN* |
| *Scarv1 + Arv1^L13A^* | ****a*** ura leu2 trp1 ade2 ARV1::Kan^r^* | *pRS416-ARV1 ^L13A^ -HA-URA3-CEN* |
| *Scarv1 + Arv1^C27A^* | ****a*** ura leu2 trp1 ade2 ARV1::Kan^r^* | *pRS416-ARV1 ^C27A^ -HA-URA3-CEN* |
| *Scarv1 + Arv1 ^C30A^* | ****a*** ura leu2 trp1 ade2 ARV1::Kan^r^* | *pRS416-ARV1 ^C30A^ -HA-URA3-CEN* |
| *Scarv1 + Arv1 ^Y37A^* | ****a*** ura leu2 trp1 ade2 ARV1::Kan^r^* | *pRS416-ARV1 ^Y37A^ -HA-URA3-CEN* |
| *Scarv1 + Arv1 ^E39D^* | ****a*** ura leu2 trp1 ade2 ARV1::Kan^r^* | *pRS416-ARV1 ^E39D^ -HA-URA3-CEN* |
| *Scarv1 + Arv1 ^I47A^* | ****a*** ura leu2 trp1 ade2 ARV1::Kan^r^* | *pRS416-ARV1 ^I47A^ -HA-URA3-CEN* |
| *Scarv1 + Arv1 ^L51A^* | ****a*** ura leu2 trp1 ade2 ARV1::Kan^r^* | *pRS416-ARV1 ^L51A^  -HA-URA3-CEN* |
| *Scarv1 + Arv1 ^N63Q^* | ****a*** ura leu2 trp1 ade2 ARV1::Kan^r^* | *pRS416-ARV1 ^N63Q^ -HA-URA3-CEN* |
| *ScARV1* | ****a*** ura leu2 trp1 ade2* | *pRS415-ERG11-MYC -URA3-CEN* |
| *Scarv1* | ****a*** ura leu2 trp1 ade2 ARV1::Kan^r^* | *pRS415-ERG11-MYC -URA3-CEN* |
| *Scarv1 + Arv1 ^C3A^* | ****a*** ura leu2 trp1 ade2 ARV1::Kan^r^* | *pRS415-ERG11-MYC -URA3-CEN* |
| *Scarv1 + Arv1 ^C27A^* | ****a*** ura leu2 trp1 ade2 ARV1::Kan^r^* | *pRS415-ERG11-MYC -URA3-CEN* |
| *Scarv1 + Arv1 ^Y37A^* | ****a*** ura leu2 trp1 ade2 ARV1::Kan^r^* | *pRS415-ERG11-MYC -URA3-CEN* |
| *ScARV1* | ****a*** ura leu2 trp1 ade2* | *pCB74-GFP-Erg11-TRP1-2u* |
| *Scarv1* | ****a*** ura leu2 trp1 ade2 ARV1::Kan^r^* | *pCB74-GFP-Erg11-TRP1-2u* |
| *Scarv1 + Arv1^C3A^* | ****a*** ura leu2 trp1 ade2 ARV1::Kan^r^* | *pCB74-GFP-Erg11-TRP1-2u* |
| *Scarv1 + Arv1 ^C27A^* | ****a*** ura leu2 trp1 ade2 ARV1::Kan^r^* | *pCB74-GFP-Erg11-TRP1-2u* |
| *Scarv1 + Arv1 ^Y37A^* | ****a*** ura leu2 trp1 ade2 ARV1::Kan^r^* | *pCB74-GFP-Erg11-TRP1-2u* |
| *ScARV1* | ****a*** ura leu2 trp1 ade2* | *pRS406-UPRE-LacZ* |
| *Scarv1* | ****a*** ura leu2 trp1 ade2 ARV1::Kan^r^* | *pRS406-UPRE-LacZ* |
| *Scarv1 + Arv1^C3A^* | ****a*** ura leu2 trp1 ade2 ARV1::Kan^r^* | *pRS406-UPRE-LacZ* |
| *Scarv1 + Arv1^C27A^* | ****a*** ura leu2 trp1 ade2 ARV1::Kan^r^* | *pRS406-UPRE-LacZ* |
| *Scarv1 + Arv1 ^Y37A^* | ****a*** ura leu2 trp1 ade2 ARV1::Kan^r^* | *pRS406-UPRE-LacZ* |
