# Supplementary material for: An Erg11 lanosterol 14-α-demethylase-Arv1 complex is required for Candida albicans virulence
Source: PLoS One. 2020 Jul 17;15(7):e0235746. doi: 10.1371/journal.pone.0235746 (PMC7367482; doi:10.1371/journal.pone.0235746)
Supplement: S2 Table — (DOCX) [file pone.0235746.s002.docx]

Table S2 *C. albicans* strains

| *C. albicans* |  |
| --- | --- |
| Strains | Genotype |
| BWP17 (WT); *CaARV1/CaARV1* | *ura3/ura3::URA3 arg4::ARG4/arg4 his1::HIS1/his1* |
| *Caarv1/Caarv1* | *ura3/ura3::URA3 arg4/arg4 his1::ARV1/his1 ARV1::ARG4 ARV1::URA3* |
| *Caarv1/Caarv1* | *ura3/ura3::URA3 arg4/arg4 his1::HIS1/his1 ARV1::ARG4 ARV1::URA3* |
| *Caarv1/Caarv1^C3A^* | *ura3/ura3::URA3 arg4/arg4 his1::ARV1^C3A^/his1 ARV1::ARG4 ARV1::URA3* |
| *Caarv1/Caarv1 ^C28A^* | *ura3/ura3::URA3 arg4/arg4 his1:: ARV1^C28A^/his1 ARV1::ARG4 ARV1::URA3* |
| *Caarv1/Caarv1 ^Y38A^* | *ura3/ura3::URA3 arg4/arg4 his1:: ARV1^Y38A^/his1 ARV1::ARG4 ARV1::URA3* |
| *CaARV1/CaARV1* | *ura3/ura3::GFP-ERG11-URA3 arg4/arg4::ARG4 his1/his1::HIS1* |
| *Caarv1/Caarv1* | *ura3/ura3:: GFP-ERG11-URA3 arg4/arg4 ARV1::ARG4 his1/his1 ARV1::HIS1* |
| *Caarv1/Caarv1^C3A^* | *ura3/ura3:: GFP-ERG11-URA3 arg4/arg4::ARG4::ARV1^C3A^ his1/his1::HIS1::ARV1^C3A^* |
| *Caarv1/Caarv1 ^C28A^* | *ura3/ura3:: GFP-ERG11-URA3 arg4/arg4::ARG4::ARV1^C28A^ his1/his1::HIS1::ARV1^C28A^* |
| *Caarv1/Caarv1 ^Y38A^* | *ura3/ura3:: GFP-ERG11-URA3 arg4/arg4::ARG4::ARV1^Y38A^ his1/his1::HIS1::ARV1^Y38A^* |
