# Supplementary figures and images for: An Erg11 lanosterol 14-α-demethylase-Arv1 complex is required for Candida albicans virulence
Source: PLoS One. 2020 Jul 17;15(7):e0235746. doi: 10.1371/journal.pone.0235746 (PMC7367482; doi:10.1371/journal.pone.0235746)

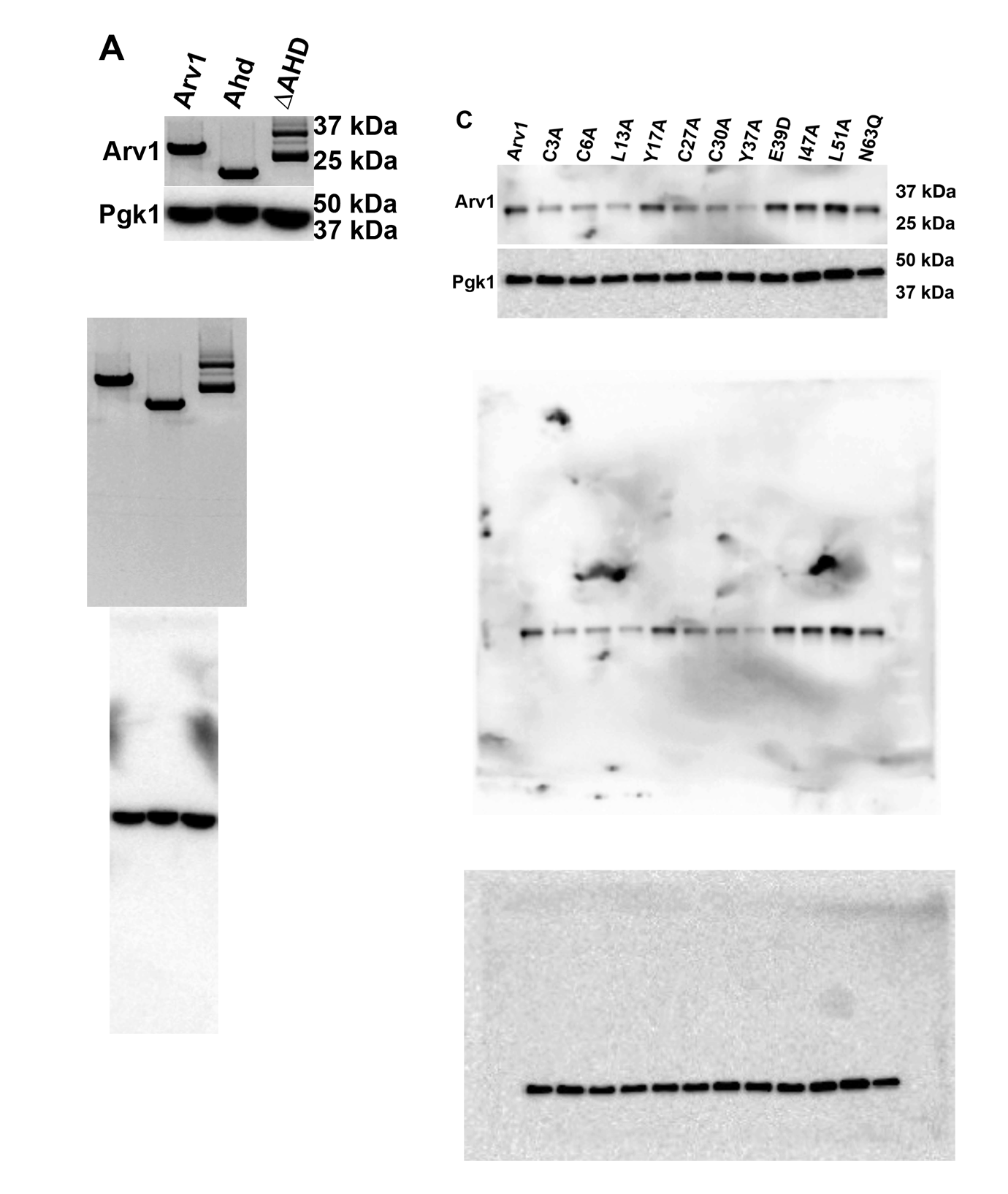

Supplement: S1 Raw image — (TIF) [file pone.0235746.s003.tif]

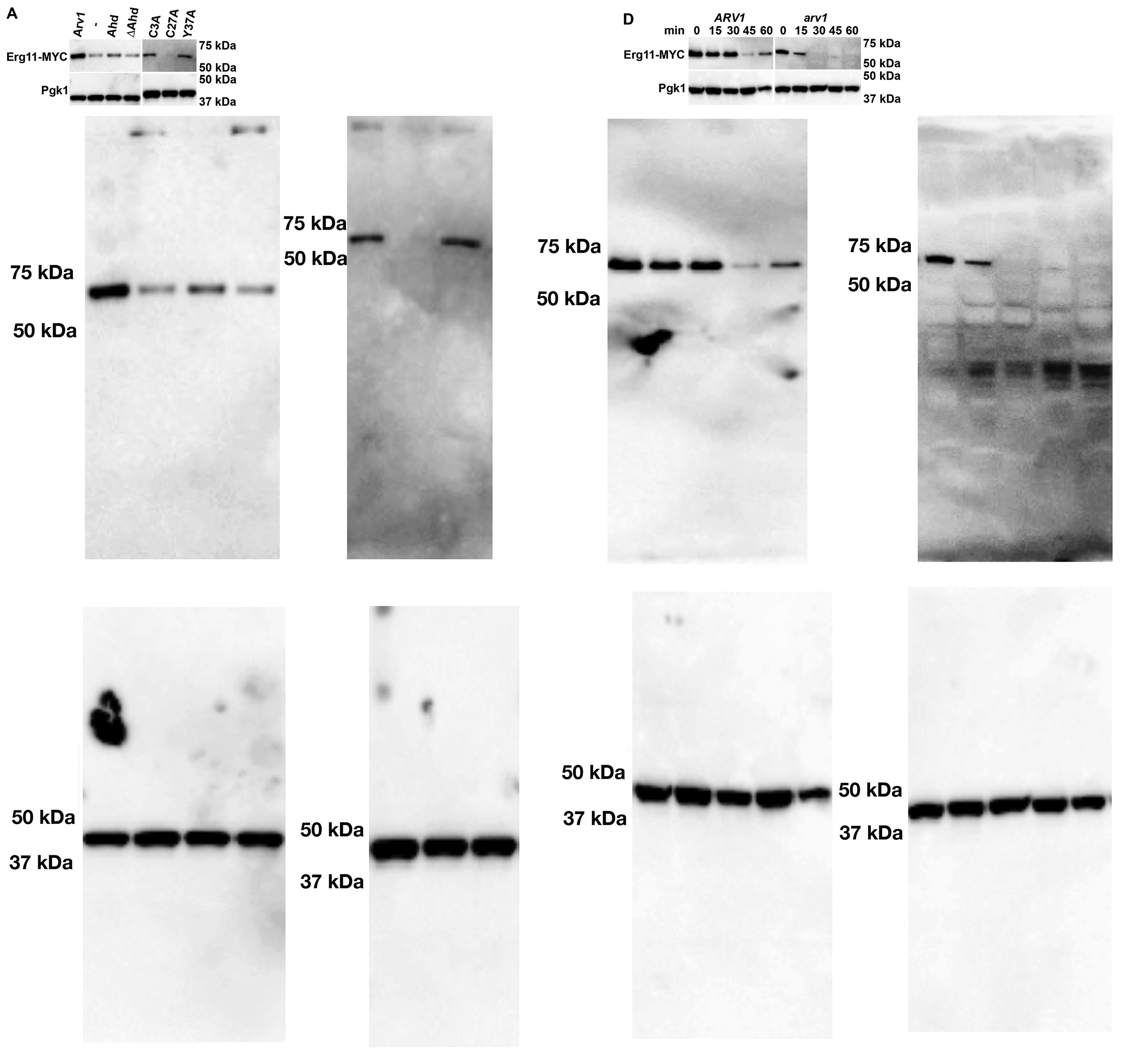

Supplement: S2 Raw image — (TIF) [file pone.0235746.s004.tif]

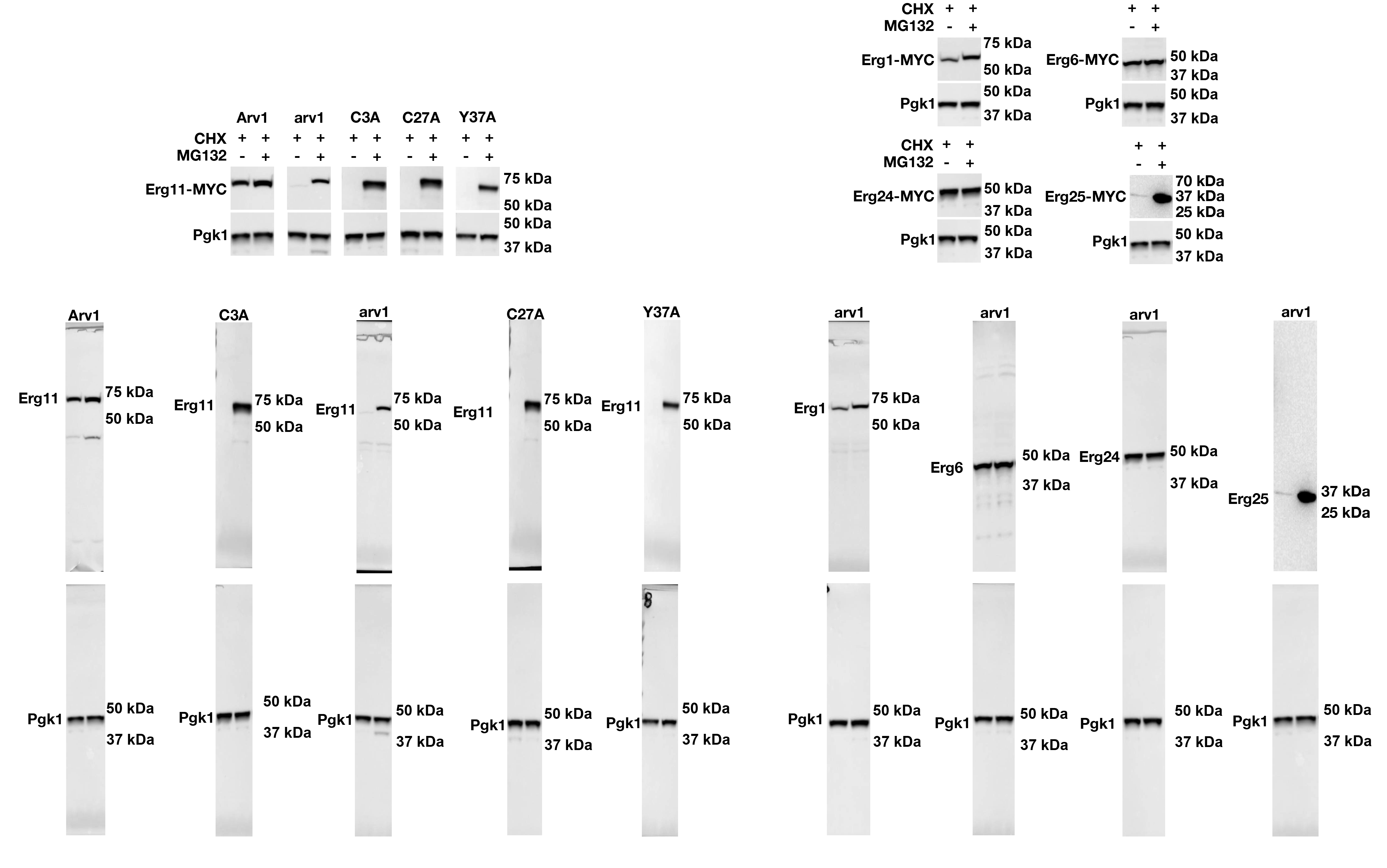

Supplement: S3 Raw image — (TIF) [file pone.0235746.s005.tif]

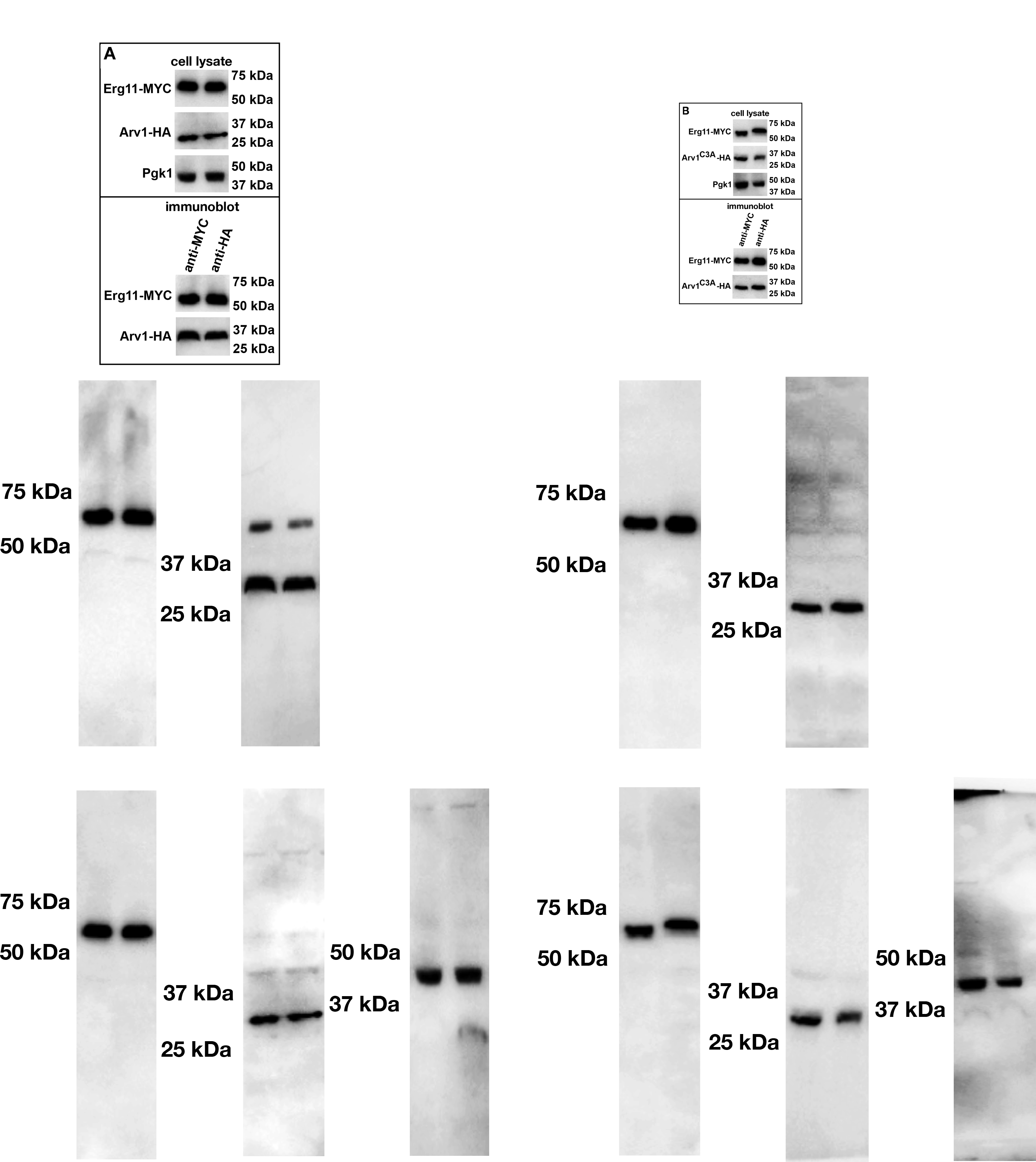

Supplement: S4 Raw image — (TIF) [file pone.0235746.s006.tif]

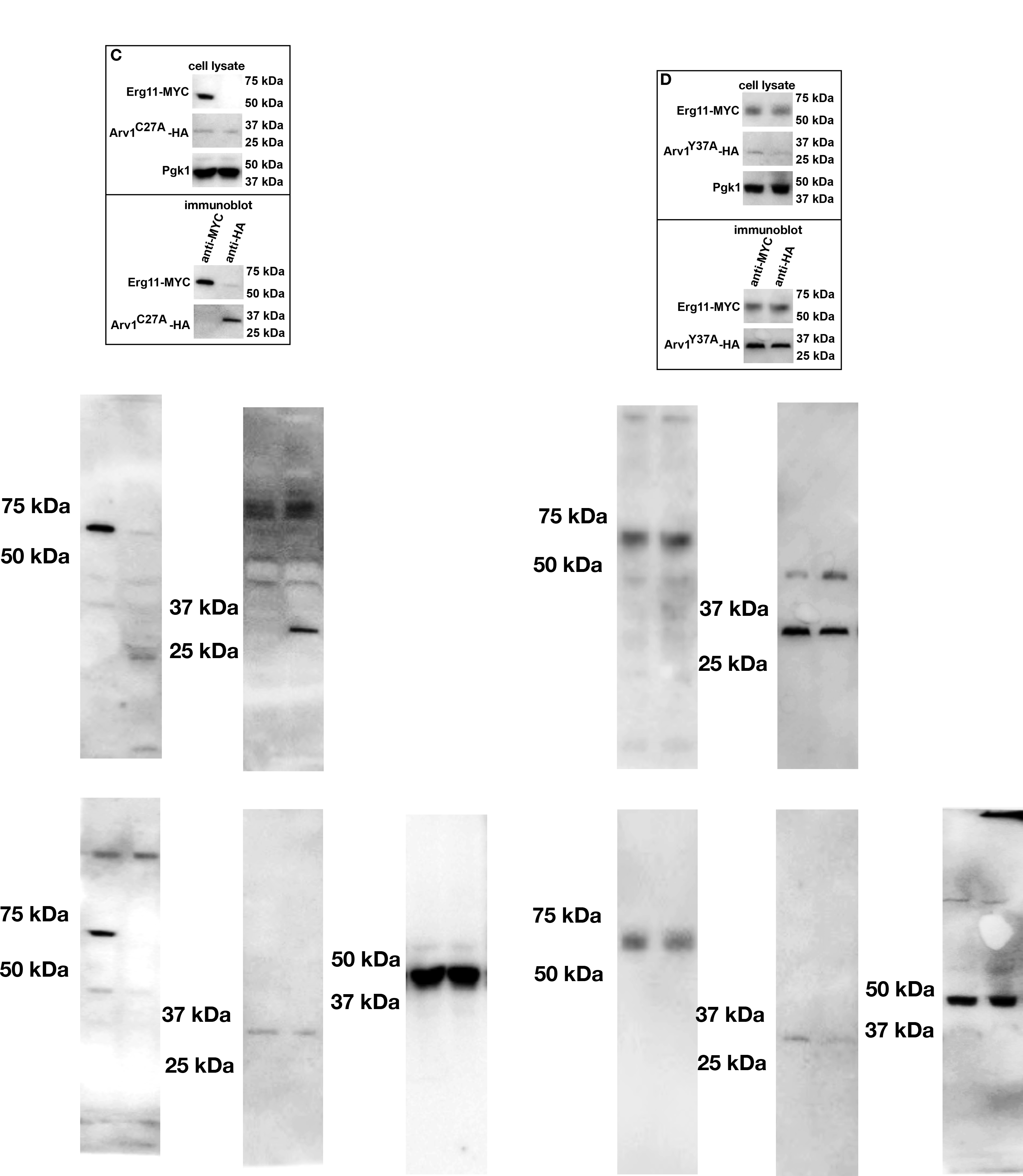

Supplement: S5 Raw image — (TIF) [file pone.0235746.s007.tif]

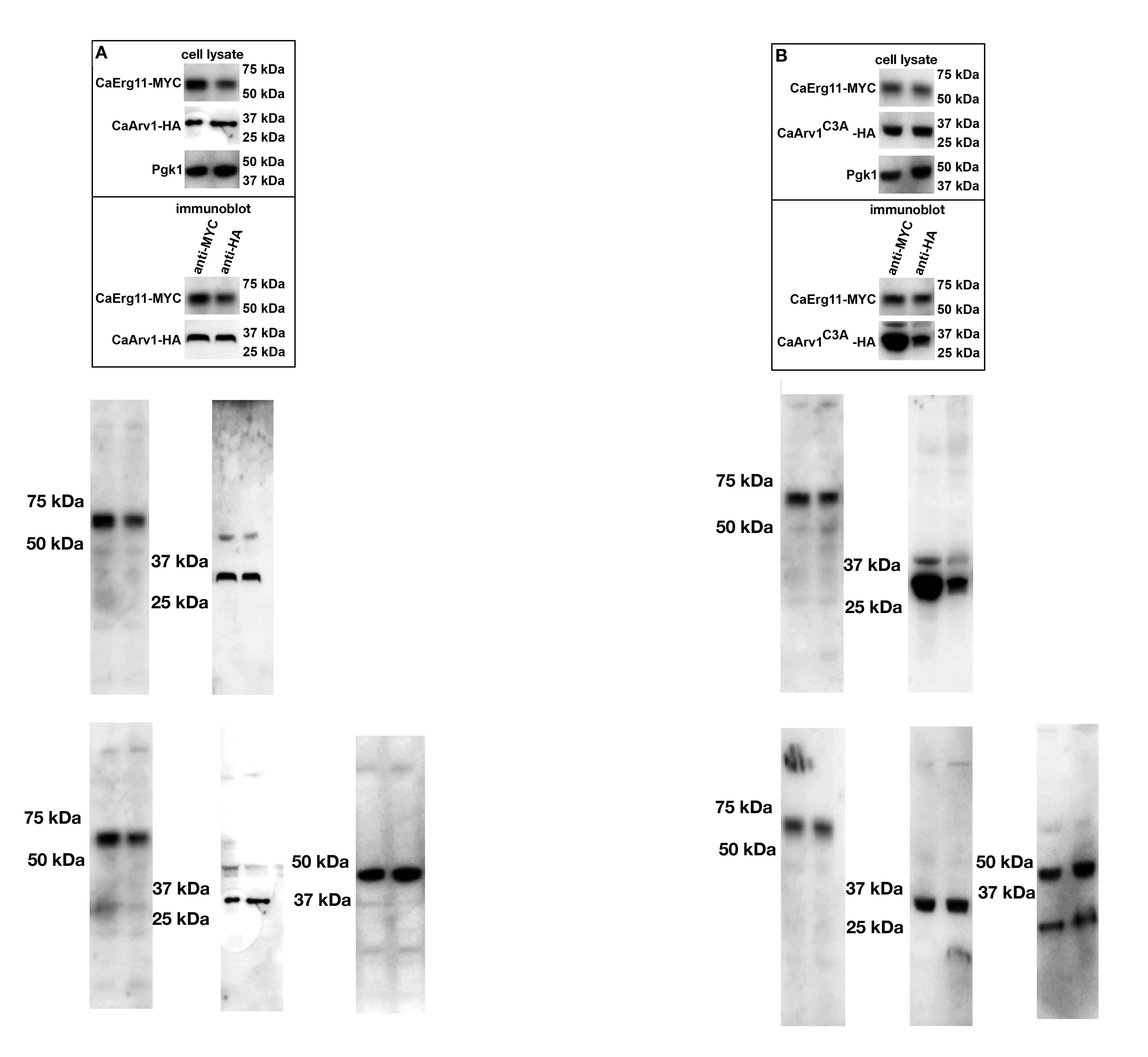

Supplement: S6 Raw image — (TIF) [file pone.0235746.s008.tif]

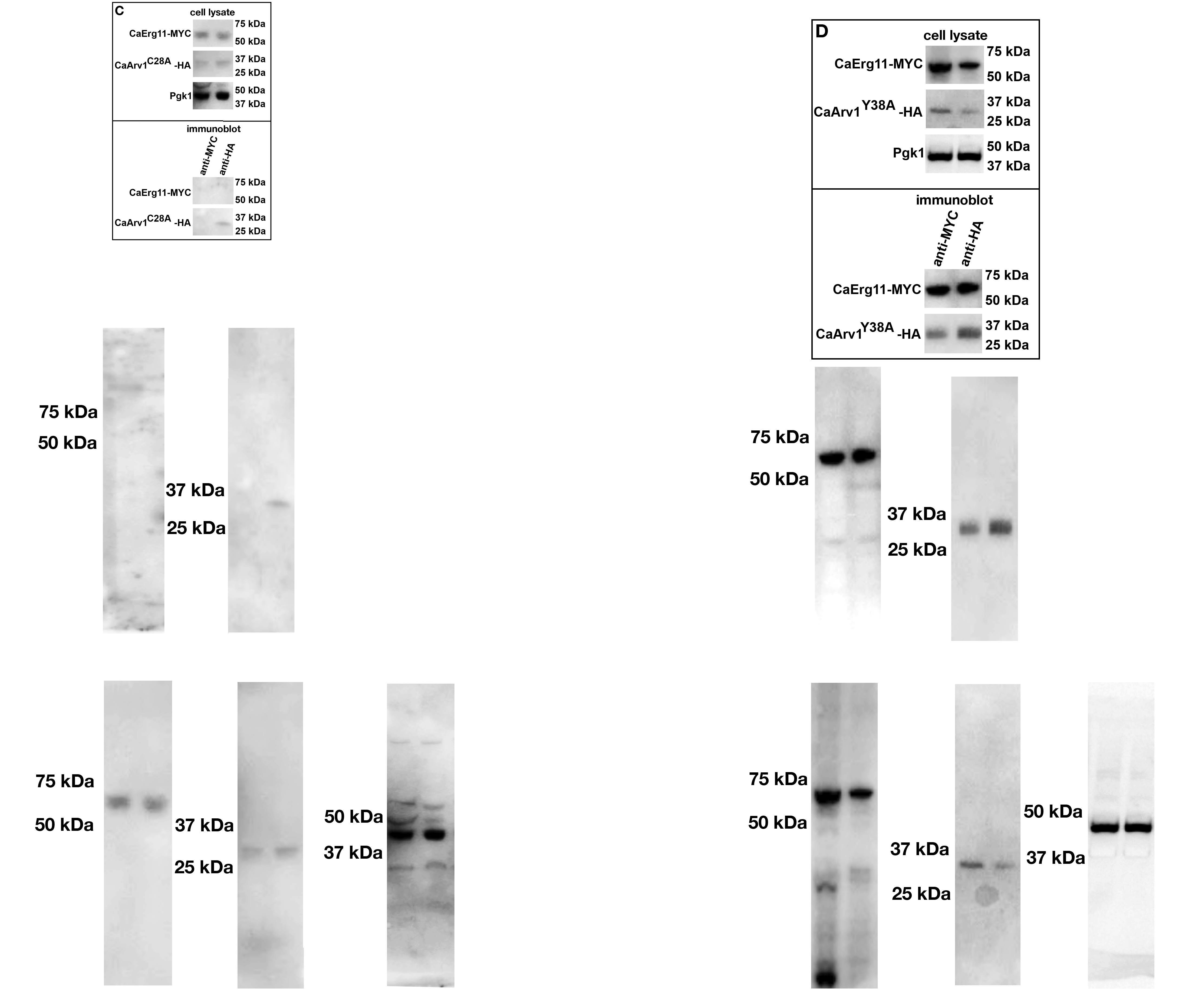

Supplement: S7 Raw image — (TIF) [file pone.0235746.s009.tif]

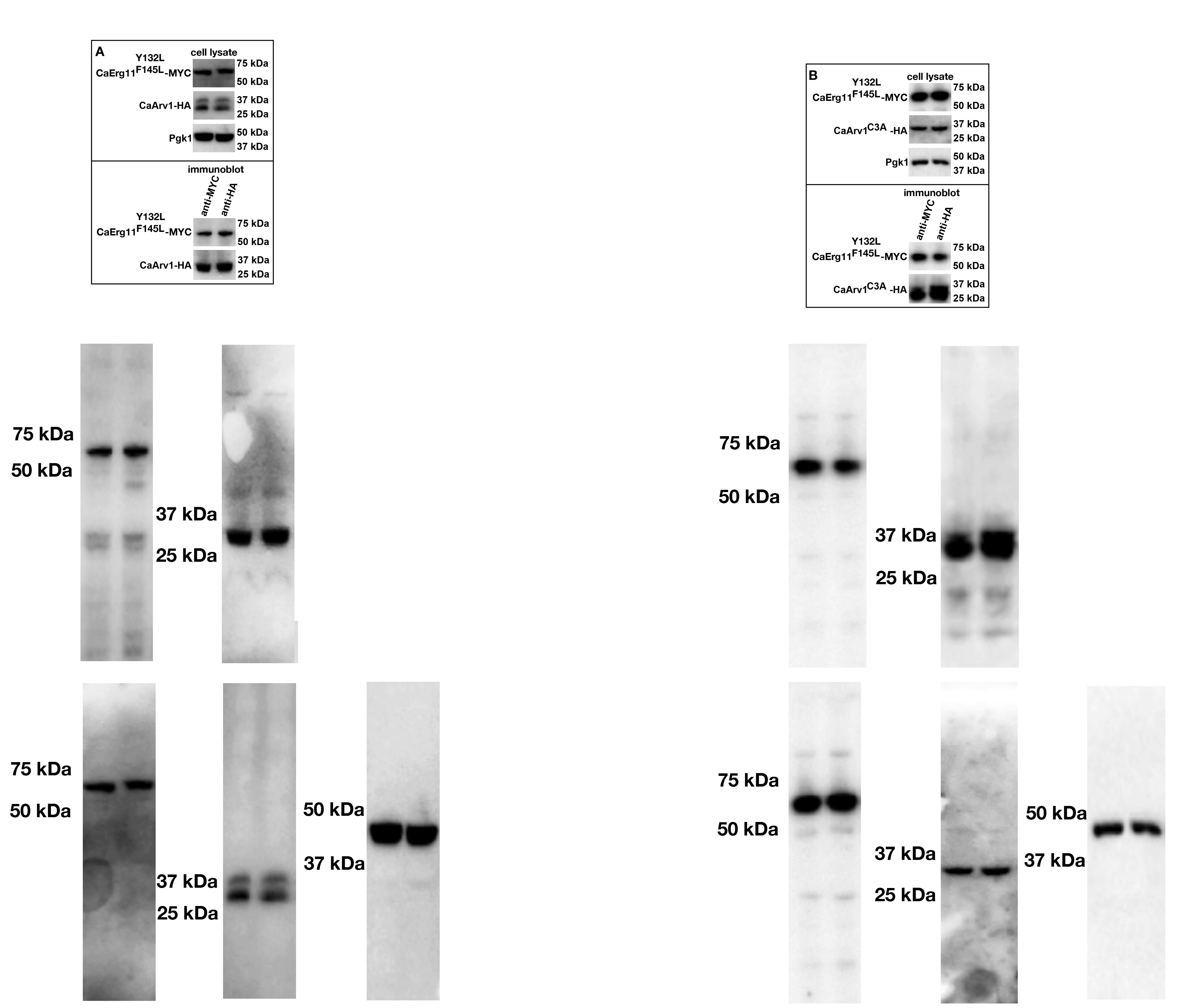

Supplement: S8 Raw image — (TIF) [file pone.0235746.s010.tif]

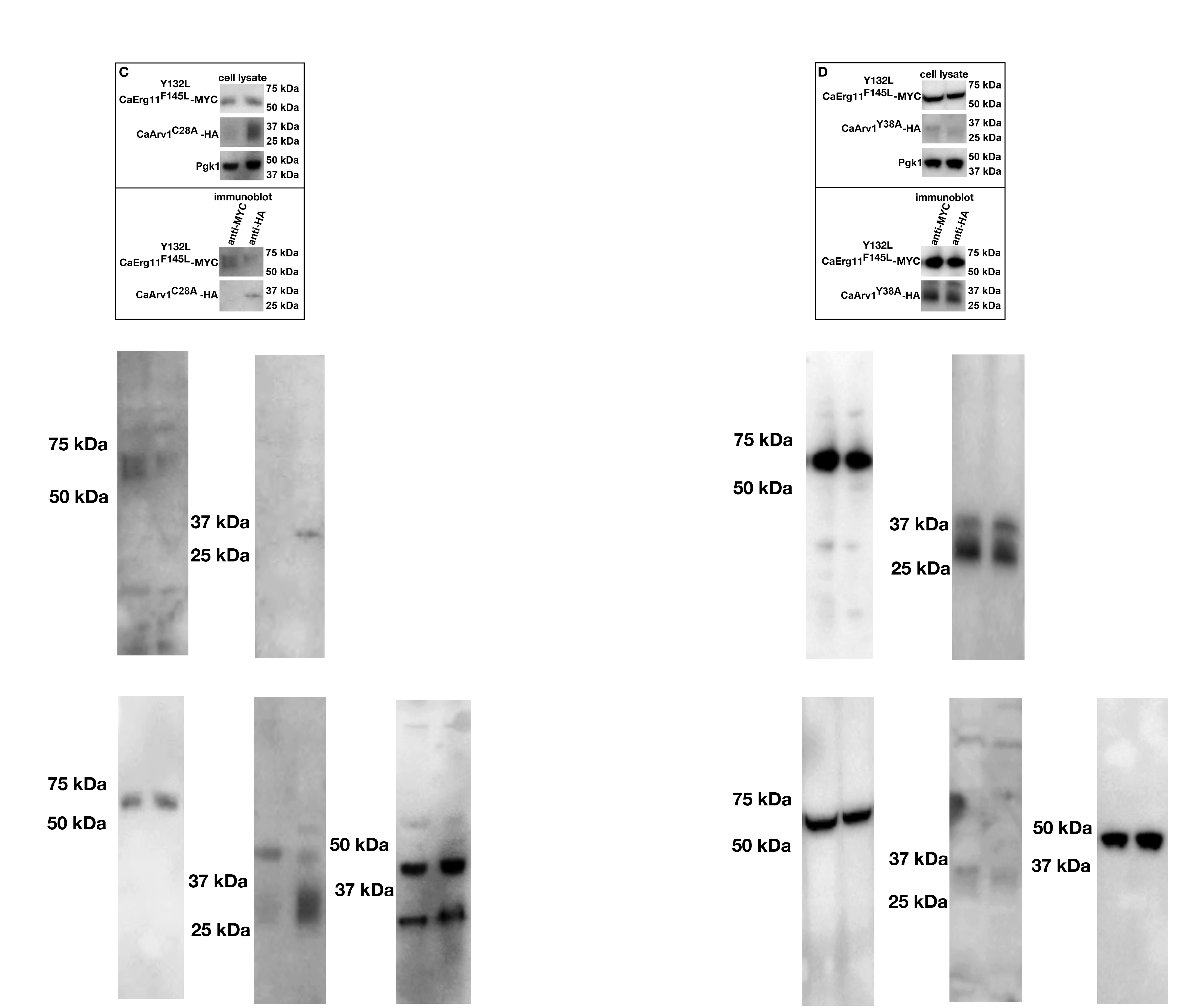

Supplement: S9 Raw image — (TIF) [file pone.0235746.s011.tif]
